# Signal peptide exchange alters HIV-1 envelope antigenicity and immunogenicity

**DOI:** 10.3389/fimmu.2024.1476924

**Published:** 2024-09-24

**Authors:** Chitra Upadhyay, Priyanka Rao, Mohammad Amin Behzadi, Roya Feyznezhad, Gregory S. Lambert, Rajnish Kumar, Madhu Kumar, Weiming Yang, Xunqing Jiang, Christina C. Luo, Arthur Nadas, James Arthos, Xiang-Peng Kong, Hui Zhang, Catarina E. Hioe, J. Andrew Duty

**Affiliations:** ^1^ Division of Infectious Diseases, Department of Medicine, Icahn School of Medicine at Mount Sinai, New York, NY, United States; ^2^ Department of Microbiology, Icahn School of Medicine at Mount Sinai, New York, NY, United States; ^3^ Department of Pathology, Johns Hopkins University, Baltimore, MD, United States; ^4^ Department of Biochemistry and Molecular Pharmacology, New York University Grossman School of Medicine, New York, NY, United States; ^5^ Department of Environment Medicine, New York University Grossman School of Medicine, New York, NY, United States; ^6^ National Institute of Allergy and Infectious Diseases, National Institutes of Health, Bethesda, MD, United States; ^7^ Research Service, James J. Peters VA Medical Center, Bronx, NY, United States

**Keywords:** HIV-1 vaccine, HIV-1 envelope, signal peptide, glycosylation, antigenicity, immunogenicity, antibody-dependent cellular phagocytosis, neutralization

## Abstract

**Introduction:**

HIV-1 envelope (Env) is the key target for antibodies (Abs) against the virus and thus an important HIV-1 vaccine component. Env is synthesized from a gp160 precursor with a signal peptide (SP) at its N-terminus. This study investigated the influence of the SP on Env antigenicity and immunogenicity.

**Methods:**

Env proteins from two HIV-1 isolates, AA05 and AC02, were analyzed as gp120 and gp160 in their native wild-type (WT) forms and as chimeras with swapped SPs (AA05-02 and AC02-05). The WT and chimeric Env were assessed for antigenicity and glycosylation using monoclonal antibodies (mAbs) and glycan probes. Immunogenicity was tested in mice using three vaccine types: gp120 protein, gp120 DNA+gp120 protein, and gp120 DNA+gp160 DNA.

**Results:**

The recombinant AC02 gp120 protein was antigenically superior to AA05 as indicated by higher reactivity with most mAbs tested. When SPs were swapped, the antigenicity of the chimeric gp120s (AA05-02 and AC02-05) resembled that of the gp120s from which the SPs were derived; AA05-02 was similar to AC02 and vice versa. Glycan probe reactivity followed a similar pattern: AA05-02 and AC02 showed similar affinity to high-mannose specific mAbs and lectins. Interestingly, the antigenicity of gp160s showed an opposite pattern; membrane-bound gp160 expressed with the AA05 SP (AA05 and AC02-05) showed greater mAb binding than gp160 with the AC02 SP (AC02 and AA05-02). Mice immunized with gp120 protein showed that AA05-02 induced stronger cross-reactive binding Ab responses than AA05 WT, and AC02 elicited stronger responses than AC02-05, indicating AC02 SP enhanced gp120 immunogenicity. However, when DNA vaccines were included (gp120 DNA+gp120 protein and gp120 DNA+gp160 DNA), the use of heterologous SPs diminished the immunogenicity of the WT immunogens. Among the three vaccine regimens tested, only gp120 DNA+gp160 DNA immunization elicited low-level Tier 2 neutralizing Abs, with AA05 WT inducing Abs with greater neutralization capabilities than AA05-02.

**Conclusion:**

These data demonstrate that the SP can significantly impact the antigenicity and immunogenicity of HIV-1 Env proteins. Hence, while SP swapping is a common practice in constructing Env immunogens, this study highlights the importance of careful consideration of the effects of replacing native SPs on the immunogenicity of Env vaccines.

## Introduction

The envelope glycoprotein (Env) of human immunodeficiency virus type 1 (HIV-1) is the key target for the development of HIV-1 vaccines. The HIV-1 Env is synthesized as a gp160 precursor protein in the endoplasmic reticulum (ER), where signal peptide (SP) cleavage, folding, addition of high-mannose glycans, and trimerization, in association with molecular chaperones, takes place (1–4). Once the nascent polypeptide attains its native folding state the SP is cleaved, and the Env egresses from the ER and translocates to the Golgi apparatus (1, 5–11). In the Golgi, gp160 is cleaved by host furin-like proteases to generate a transmembrane gp41 subunit and a non-covalently associated surface gp120 subunit. Three gp120-gp41 heterodimers assemble to form the trimeric functional Env spikes that are then directed to the plasma membrane for incorporation into virions (1, 9–11). The gp120 subunit is the primary viral component that is exposed on the virion surface and is the key target for neutralizing antibodies (12).

The HIV-1 Env has evolved a variety of mechanisms to evade host antibody (Ab) responses, including tolerance to escape mutations in immunogenic epitopes (13, 14), extensive glycosylation (15–18), conformational masking (19), mimicry of host proteins (20), and low expression of Env on virus-infected cells and virions (21, 22). Together, these features have posed substantial barriers to the development of effective Env-based vaccines. Of the many clinical vaccine trials conducted to date, RV144 is the only one that showed a modest efficacy, 59.9% at 1 year and 31.2% at 3.5 years post-vaccination (23–25). The trial did not elicit broadly neutralizing antibodies (bNAbs); rather, non-neutralizing Abs (nNAbs) against the V1V2 and Ab-dependent Fc-effector functions were identified as the correlates of protection (26–30). Epitopes of the V1V2 Abs have been grouped into at least three types: V2i, V2p, and V2q (31, 32). RV144 vaccinees produced V2p- and V2i-type Abs, but not the V2q-type Abs (24, 28). The trial tested the “prime-boost” combination of two vaccines: ALVAC^®^ HIV vaccine (prime) and AIDSVAX^®^ B/E vaccine (boost). The ALVAC^®^-HIV utilized a recombinant canarypox vector to express HIV-1 LAI Gag and Pol alongside monomeric 92TH023 gp120 linked to the transmembrane anchoring portion of LAI gp41. The SP of HIV-1 Env is known to be responsible for poor expression and low secretion of Env (33). To achieve higher expression of Env protein immunogens, replacing the Env SP with non-HIV-1 SPs is a common strategy practiced in the HIV-1 vaccine field to increase Env protein yields. The AIDSVAX^®^ B/E in RV144 was a formulation of recombinant Env gp120 proteins from MN and A244 strains wherein the first 11 amino acids of Env and the native SP were replaced with a 27 amino acid sequence from HSV-1 gD (23, 34). The other most common SP used for driving the increased secretion of protein immunogens is the SP from tissue plasminogen activator (tPA) protein, which is used for the production of soluble trimeric Env mimics, e.g. SOSIP (35, 36). The tPA SP is also a preferred choice for replacing the native SP for immunogens for other pathogens including dengue and influenza (37–43).

Signal peptides (SPs) generally consist of 16 to 30 amino acids and are characterized by a tripartite structure: a hydrophilic, positively charged N-terminal region, a hydrophobic central H-region, and a slightly polar C-terminal region with a cleavage site for signal peptidase (44). The SP of HIV-1 Env, which includes the initial ~30 amino acids of the gp160 protein, exhibits significant sequence variability among different HIV-1 strains and has a striking number of charged residues in its N-terminal region (45, 46). Another unique feature of the HIV-1 Env SP is its post-translational cleavage (6, 7, 47–50), allowing a longer retention time for the Env protein in the ER (3, 8, 33, 51–53). Despite the diversity in SP sequences, this delayed cleavage is a conserved process across HIV-1 variants and is critical for folding the native Env with great fidelity (3, 33, 52, 54). Therefore, the delayed cleavage of the gp160 SP acts as a crucial quality control and folding facilitator (6, 50). Our published research also has established the impact of SP on the glycosylation of HIV-1 Env and the resulting viral phenotype (45, 55–57). We have demonstrated that even a single mutation in the SP can significantly modify the composition and structure of the glycans on Env, influencing its interaction with anti-HIV-1 Abs and DC-SIGN mediated trans-infection (45, 58–60). Swapping the SP from one HIV-1 strain with another can lead to even more pronounced changes in Env glycosylation and its properties, including antigenicity, neutralization sensitivity, and transmission efficiency (55, 56).

Comparison of SP sequences from Env of acute versus chronic HIV-1 isolates identified a signature amino acid that associated with the transition from acute to chronic stages; histidine at position 12 (H12) is enriched in SPs from acute isolates and absent in those from chronic isolates (47, 48). A similar pattern was also observed in the SPs of simian immunodeficiency virus (SIV) Env (49). The presence of this signature motif suggests a potential role of SP in regulating Env phenotypes at different stages of infection. Indeed, we demonstrated that mutating H12 to three different amino acids (H12R, H12Y and H12Q) affected the levels of Env incorporated into virions which was associated with changes in virus infectivity and transmission. Additionally, analogous SP mutations altered Env glycosylation and virus vulnerability to neutralization by Env-specific mAbs (45).

In this study, we aimed to assess how the Env SP influences the antigenic and immunogenic properties of the clade B AA05 and AC02 Env proteins. Both strains have been studied previously (61), exhibit unique antigenic characteristics, and differ by six residues including the H12 signature motif; AA05 contains the H12 residue in its SP, which is missing in AC02 SP (47–49). Specifically, we evaluated the impact of exchanging SPs on the antigenicity and immunogenicity of Env. We tested the effects of SP exchange on Env immunogenicity in mice using three vaccine regimens: gp120 protein, gp120 DNA+gp120 protein, and gp120 DNA+gp160 DNA. Vaccine-induced serum Abs were evaluated against a panel of HIV-1 Env antigens to detect cross-reactive binding Ab responses and tested for neutralization and antibody-dependent cellular phagocytosis (ADCP) activities. The findings highlight the regulatory role of SP in shaping the capacity of Env to elicit binding and functional antibodies against HIV-1.

## Material and methods

### Cell lines

HEK293T/17 cells (293T) were obtained from the American Type Culture Collection (ATCC) and were used to produce infectious HIV-1 viruses. TZM.bl cell line was obtained through the NIH AIDS Reagent Program, Division of AIDS, NIAID, NIH, contributed by Dr. John C. Kappes, Dr. Xiaoyun Wu and Tranzyme Inc (62). The TZM.bl cell line is a derivativative of HeLa cells modified to express HIV-1 receptor (CD4), co-receptors (CCR5 and CXCR4), and contains reporter cassettes of luciferase and β-galactosidase that are each expressed from an HIV-1 LTR. The TZM.bl cell line was used to assay virus infectivity and virus neutralization. The 293T and TZM.bl cell lines were routinely subcultured every 3-4 days by trypsinization and were maintained in Dulbecco’s modified Eagle’s medium (DMEM) supplemented with 10% heat-inactivated fetal bovine serum (FBS), HEPES pH 7.4 (10 mM), L-glutamine (2 mM), penicillin (100 U/ml), and streptomycin (100 μg/ml). Mouse myoblast cell line C2C12 was obtained from ATCC and was maintained in the ATCC-formulated Dulbecco’s Modified Eagle’s Medium supplemented with 10% FBS. The C2C12 cell line was used to test the antigenicity of gp160 plasmids mimicking the expression of Env in mouse muscle cells. All cell lines were maintained at 37°C in a humidified atmosphere with 5% CO2. ExpiCHO-S cells (Thermo Fisher Scientific) were maintained ExpiCHO expression media with gentle shaking at 37°C in the presence of 8% CO2 as per the manufactuer’s instructions. ExpiCHO-S cells were used to express recombinant gp120 proteins.

### DNA plasmids and recombinant gp120 proteins

**Figure 1:**
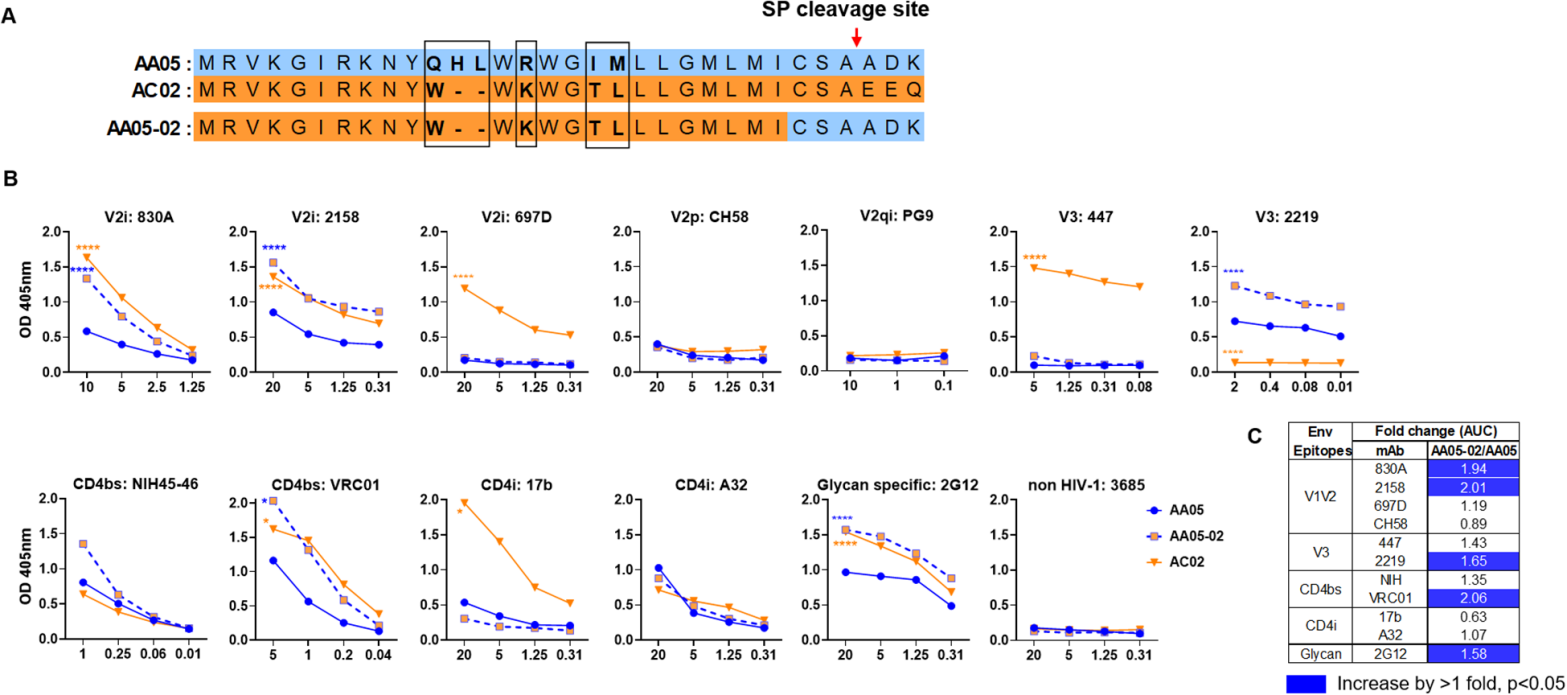
Changes in antigenicity of AA05 gp120 upon signal peptide (SP) swapping. **(A)** Schematic representation of WT and SP swapped proteins evaluated in this study. Dashes (-) indicate missing residues. Differences between the SP are encased and shown in bold. **(B)** WT and SP swapped gp120 proteins were coated onto ELISA plates (2 ug/ml) and reacted with titrated amounts of antibodies. An irrelevant non-HIV-1 mAb specific for anthrax (3685) was used as negative control. AA05-02 and AC02 were compared to AA05 gp120 protein. *p<0.05; ****p<0.0001 by 2-way ANOVA. p>0.05 was left unmarked. **(C)** Summary of the changes in antigenicity upon SP swapping. Fold changes of SP swapped/WT were calculated for each mAb tested from **(B)**. Significant increases are marked with blue (fold change of >1 with p<0.05).

**Figure 2:**
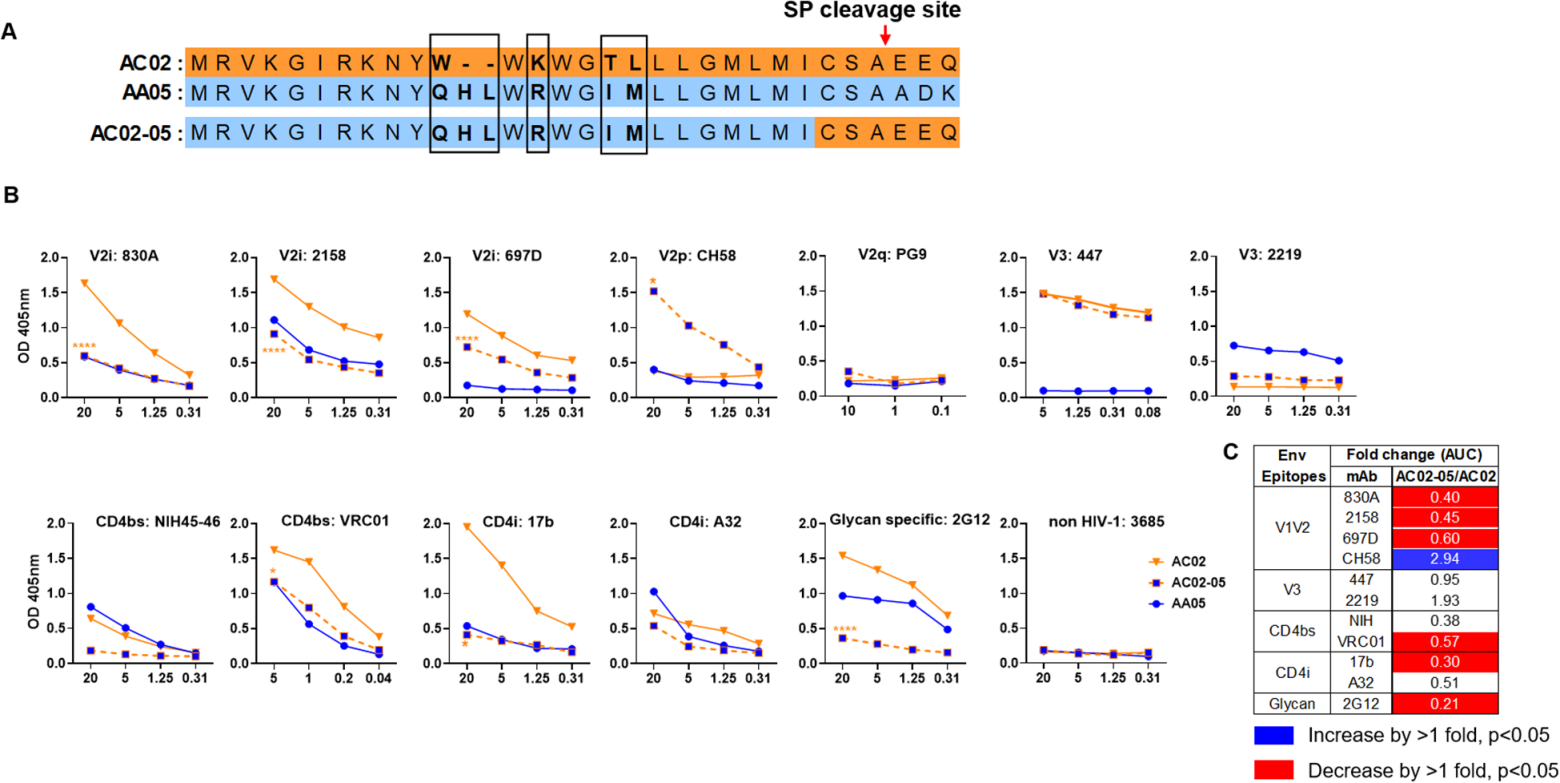
Changes in antigenicity of AC02 gp120 upon signal peptide (SP) swapping. **(A)** Schematic representation of WT and SP swapped proteins evaluated in this study. Dashes **(-)** indicate missing residues. Differences between the SP are encased and shown in bold. **(B)** WT and SP swapped gp120 were coated onto ELISA plates (2 ug/ml) and reacted with titrated amounts of antibodies. An irrelevant non-HIV-1 mAb specific for anthrax (3685) was used as negative control. AC02-05 was compared to AC02. *p<0.05; ****p<0.0001 by 2-way ANOVA. p>0.05 was left unmarked. **(C)** Summary of the changes in antigenicity upon SP swapping. Fold changes (AUC) of SP swapped/WT were calculated for each mAb tested from **(B)**. Significant increases or decreases are marked with blue (fold change of >1 with p<0.05) or red (fold change of <1 with p<0.05), respectively.

The gp120-encoding DNA plasmids were constructed with the pCAGGS vector. The gp120 proteins were expressed under native (AA05, AC02) or heterologous SP (AA05-02, AC02-05) (**Figures 1A**, **2A**). Similarly, WT and SP swapped DNA plasmids encoding full length gp160 were also constructed. The plasmids were purified using Invitrogen PureLink Expi Endotoxin-Free Plasmid Purification kits (Thermo Fisher Scientific). The gp120 plasmids were used to produce recombinant protein vaccines as described below and tested as gp120 DNA vaccines in the mouse co-immunization experiment. A mixture of gp120 and gp160 plasmids in 1:1 ratio was also tested as DNA immunogens.

The WT and SP- swapped recombinant gp120 proteins were produced by transient transfection of ExpiCHO-S cells using the ExpiCHO Expression System Max Titer Protocol (Thermo Fisher Scientific). Transfected cells were cultured in ExpiCHO expression media for 10-14 days with gentle shaking at 37°C in the presence of 8% CO2, after which the supernatants were collected by centrifugation at 300 x g for 15 min at 4°C to remove cells, followed by a second centrifugation at 5,000 x g for 20 min at 4°C, and a 0.2 μm filtration to remove cellular debris. Supernatants were then applied to a Galanthus nivalis lectin column (Vector Labs) and eluted with 200 mM α-methyl-mannopyranoside (Sigma), desalted to PBS, and passed through a Superdex-200 16/60 gel-filtration column (GE Healthcare Bio-Sciences). Peak fractions were collected, checked on a 12% SDS-PAGE reducing gel, and concentrated using a 10kDa cut-off filter (Millipore). Proteins were quantified by UV absorbance at 280 nm and verified for anti-Env antibody recognition by ELISA and Western blot analysis.

### Antibodies and lectins

The following Ab reagents used in this study were obtained through the NIH AIDS Reagent Program, Division of AIDS, NIAID, NIH: anti-HIV-1 gp120 mAbs CH58 and CH59 from Drs. Barton F. Haynes and Hua-Xin Liao (63); anti-HIV-1 gp120 mAb VRC01 from Dr. John Mascola (64); anti-HIV-1 gp120 mAb NIH45-46 from Dr. Pamela Bjorkman (65); anti-HIV-1 gp120 mAbs PG9, PG16, PGT145 from IAVI (66); anti-HIV-1 gp120 mAb A32 contributed by Dr. James Robinson (67); anti-HIV-1 gp41/gp120 mAb 35O22, from Drs. Jinghe Huang and Mark Connors (68); anti-HIV-1 gp120 mAb 2G12 and anti-HIV-1 gp41 monoclonal 2F5 contributed by DAIDS/NIAID (69); anti-HIV-1 gp41/gp120 monoclonal PGT151 from Dr Dennis Burton. The V2i and V3 mAbs, and negative control anti-anthrax mAb 3685 and anti-parvovirus mAb 1418 were obtained from the laboratory of Dr. Susan Zolla-Pazner (70–78). The lectins Galanthus nivalis (GNA), Narcissus pseudonarcissus (NPL), Aleuria aurantia (AAL) and Sambucus nigra (SNA) were purchased from Vector Laboratories.

### ELISA to test gp120 antigenicity

ELISA was performed to assess the antigenic changes induced by SP swapping. The gp120 proteins were coated onto 96-well half-area Costar plates overnight at 4°C at 2 μg/ml in PBS. Wells were subsequently washed three times with a wash buffer (PBS containing 0.1% Tween-20) using a TECAN hydro-speed plate washer and the uncoated surfaces were blocked with 150 μl of blocking buffer (RPMI 1640 Medium and 10% Fetal Bovine Serum [RPMI-10]) for 1 h at 37°C. MAbs specific for different Env epitopes were serially diluted in blocking buffer and 50 μl was added to each well. The plates were incubated for 1 h at 37°C followed by further incubation for 30 min at 37°C with secondary Ab (alkaline phosphatase (AP)-conjugated goat anti-human IgG (Sigma) at 1:1000-fold dilution in blocking buffer). The AP reaction was initiated by adding 50 μl 4-nitrophenyl phosphate disodium salt hexahydrate substrate (Sigma) in diethanolamine (Sigma). Plates were read on a microplate reader (Cytek Powerwave) at 405 nm. Data are reported as optical density 405 (OD405). Plates were washed 3x with the wash buffer after each step as described above. For lectin binding experiments, ELISA was performed as above using biotinylated lectins that were incubated with the proteins coated on the plates followed by Streptavidin-AP (Sigma). Plates were read and data are reported as optical density 405 (OD405), as above.

### Flow cytometry assay to test gp160 antigenicity

To assess the *in vitro* expression and antigenicity of the full length gp160 we used the mouse myoblast C2C12 cell line. Briefly, a total of 4 × 10^6^ C2C12 cells were seeded in 15 ml of culture medium in a 100-mm tissue culture dish and incubated at 37°C. Twenty four hours later, cells in each dish were transfected with 20 μg gp160 expression plasmid (WT or SP swaps) using TransfeX™ Transfection Reagent (ATCC) following the manufacturer’s instructions. Transfected cells were incubated for 24 hours at 37°C, washed with PBS, detached with trypsin-free cell-dissociation buffer, and resuspended in PBS containing 2% BSA. Cells were stained with LIVE/DEAD Aqua stain (Invitrogen) and distributed into 96-well V-bottom tissue culture plates (5x10^4^/well) for individual staining reactions. Cells were incubated with mAbs at the following concentrations: 830A, 2158, 697D, PG9, PG16, PGT145, 447, 2219, PGT151, A32, 2G12, 2F5, 3685 at 100 μg/ml; CH59, VRC01, 35O22 at 50 μg/ml; and NIH45-46 at 20 μg/ml. For detection of mAb binding, biotinylated goat anti-Human IgG Fc (1:1000) followed by streptavidin phycoerythrin (PE) (1:500) was used. Each mAb was tested in duplicates. The cells were washed 3X with PBS-B (PBS plus 1% BSA) after each step and all incubation steps were performed on ice for 30 min. Cells were analyzed with Attune flow cytometer, and 30,000 events were collected in the PE+ gate. Analysis was carried out using FCS-Express software as follows: C2C12 cells were selected from a plot of forward-area vs. side scatter-area (FSC-A/SSC-A) from which doublets were excluded in a forward scatter height vs forward scatter area plot (FSC-H/FSC-A). Live cells were selected by Aqua-negative gating, and geometric mean fluorescent intensity (MFI) of PE+ cells, representing anti-Env-stained cells, were quantified. Background MFI, as determined from cells stained without primary antibodies was subtracted from all Env-mAb pairs. Non-HIV-1 mAb 3685 (anti-anthrax mAb) was used as a negative control.

### Liquid chromatography mass spectrometry

Analysis for site-specific glycosylation was performed as described previously (79). Briefly, the purified gp120 proteins were denatured by dithiothreitol (10mM) and alkylated by iodoacetamide (55 mM) in ammonium bicarbonate (25 mM). The resulting proteins were digested with a combination of trypsin and chymotrypsin at an enzyme/substrate ratio of 1:15 (w/w) and 1:10 (w/w), respectively, in 25 mM ammonium bicarbonate. Sequential treatment with two endoglycosidases was then performed to introduce novel mass signatures for peptides that contain glycans of high-mannose types and complex-type glycans (79). First, the gp120 peptides were digested with enzyme Endo H to cleave the high-mannose (and hybrid) glycans between the innermost GlcNAc residues, leaving a GlcNAc attached to Asn, resulting in a shift of N+203. The subsequent treatment with PNGase F enzyme removed the remaining complex-type glycans, and in the process converted Asn to Asp, resulting in a +0.984 Da mass shift (N+1) (80, 81). For peptides with unoccupied glycosites, these treatments produce no mass shift (N+0). Using this strategy, liquid chromatography–mass spectrometry (LC-MS/MS) data were acquired for each sample. Peptides were identified using SEQUEST. Abundance of each peptide was determined by the sum of the peak areas from all identified charge states (15).

Analysis of the samples was done on a Q-Exactive mass spectrometer. Briefly, the de-glycosylated peptides were separated on a Dionex Ultimate 3000 RSLC nano system with a 75 µm × 15 cm Acclaim PepMap100 separating column protected by a 2-cm guard column. The flow rate was set at 300 nl/min and Buffer A-3% ACN (0.1% FA) and buffer B-90% ACN (0.1% FA) were used. A 130-minute gradient was run, following the steps: 0-10 min, 2-5% B; 10-90 min, 5-25% B; 90-112 min, 25-35% B; 112-115 min, 35-95% B; 115-125 min, hold at 95% B; 125-129 min, 95-2% B; 129-130 min, hold at 2% B. The Orbitrap MS1 spectra (AGC 3e6) were collected from 400–1,800 m/z at a resolution of 70 K followed by data dependent higher-energy collisional dissociation tandem mass spectrometry (HCD MS/MS) (resolution 35,000 and collision energy 31%) of the 12 most abundant ions using an isolation width of 1.4 Da. Dynamic exclusion time was set at 30s.

### Animal immunization

Protocols for animal immunizations were approved by the Institutional Animal Care and Use Committee (IACUC) of the Icahn School of Medicine at Mount Sinai. All efforts were made to minimize animal suffering. BALB/c mice (female, >6 weeks old, 5 animals per group) were used in experiments to test protein alone, DNA plus protein, and DNA alone immunizations.

For protein immunizations, mice were injected subcutaneously with gp120 proteins (3 μg gp120 per dose) after addition of adjuvant: 25 μg monophosphoryl lipid A (MPL; Sigma) and 250 μg dimethyldioctadecylammonium (DDA; Sigma) in 100 μl per dose. For negative control, animals received 100 μl PBS in MPL/DDA at each immunization. Animals were immunized 3 times at 3 week-intervals. Blood was collected 2 weeks after the last immunization and used for analysis.

For DNA plus protein co-immunization, mice were anesthetized using 4 parts ketamine HCl (100 mg/ml stock solution) to 1-part xylazine (20 mg/ml stock). Each mouse received 100 µg of gp120 DNA vaccine in PBS at each time point (50 µg in each left and right thigh) using AgilePulse *In Vivo* Electroporation System (Harvard BTX, Harvard Biosciences, Inc.) using manufacturer supplied pulse dynamics for mice/small animals. Protein immunogens were given in MPL/DDA as above at the same time points. The pre-bleed sera served as negative control for this set of experiments. Blood was collected 2 weeks after the last immunization and used for analysis.

For DNA immunizations, mice were anesthetized using 4 parts ketamine HCl (100 mg/ml stock solution) to 1 part xylazine (20 mg/ml stock). DNA vaccine comprised a mix of gp120 and gp160 expressing plasmids (1:1). Each mouse received 100 µg of DNA vaccine in PBS at each time point (50ug in each left and right thigh) using a TriGrid electroporation device (Ichor Medical Systems). The pre-bleed sera served as negative control for this set of experiments. Blood was collected 2 weeks after the last immunization and used for analysis.

### Assays to measure serum antibody levels

To measure the relative levels of mouse IgG reactive to autologous and heterologous HIV-1 Env, Luminex multiplex assays were performed using a panel of antigens including BG505.SOSIP, gp140s, gp120s, V1V2 on scaffolds and V3 peptides (82). The following reagent was obtained through the NIH HIV Reagent Program, Division of AIDS, NIAID, NIH: recombinant proteins AE.A244 delta11 gp120 (ARP-12569), C.1086 gp140C (ARP-12581), C.1086 gp140C K160N (ARP-12580), B.JRFL gp140CF (ARP-12573), UG21 gp140 (ARP-12065), CN54 gp120 (ARP-13354), B.MN delta11 gp120 (ARP-12570), RSC3 (ARP-12042) were contributed by Drs. Zhi-Yong Yang, Peter Kwong, Gary Nabel, and John Mascola, C.1086 V1V2-tags (ARP-12568) was contributed by Drs. Barton F. Haynes and Hua-Xin Liao. V1V2 (YU2 and ZM109) on 1FD6 scaffolds, AA05, AA05-02, AC02 and AC02-05 proteins were produced in the lab of Dr Xiang-Peng Kong (83, 84). YU2, HXB2, REJO and ZM109 gp120 were purchased from Immunetech.

Briefly, antigens were coupled to magnetic carboxylated fluorescent beads (Luminex Corp) using a xMAP Antibody Coupling (AbC) Kit (Luminex). Mouse serum was serially diluted (4-fold, starting with 1:100 dilution) and IgG binding to the beads was detected using anti-mouse IgG (Southern Biotech) followed by Streptavidin-PE. Samples were acquired on a Bio-Plex array reader (FlexMAP 3D, Luminex). Median fluorescence intensities (MFI) were reported for each bead type.

For measuring serum IgG binding to RSC3 core protein, ELISA were performed as described above with modifications mentioned below. Mouse serum samples, 4-fold serially diluted starting from 1:100, were added to the antigen (2 μg/ml) coated plates (25 μl/well) and incubated at 37°C for 1 hour. Subsequently, the plates were incubated with 25 μl/well of anti-mouse AP conjugated IgG (Sigma) at 1:1000 at 37°C for 30 minutes. All samples and secondary antibodies were diluted in RPMI-10 and plates were washed (5X) between each step. Finally, 4-nitrophenyl phosphate disodium salt hexahydrate substrate in diethanolamine (Sigma) was added, and OD405 was measured.

### HIV-1 neutralization

Virus neutralization was measured using HIV-1 pseudoviruses with TZM-bl target cells as described previously (19). The pseudoviruses 398F1, TRO.11, and CH119 were produced by transfecting 293T cells with pNL4.3ΔEnv backbone and gp160-expressing plasmids (1:1 ratio) using PEI (Sigma). The infectious virus RHPA was produced by transfecting 293T cells with the pRHPA infectious molecular clone. Heat-inactivated sera (56°C for 30 min) were used for testing the neutralization activity. Briefly, serially diluted sera were incubated with pseudoviruses for 1 h at 37°C and the mixtures were added to the TZM-bl cells in the presence of DEAE (Sigma). Virus infection was determined after 48 h using the Beta-Glo Luciferase Assay System (Promega).

### Antibody-dependent cellular phagocytosis

Measurement of ADCP was performed as described (85), using human THP-1 cells and FITC labelled carboxylated microspheres (1-μm diameter; ThermoFisher Scientific). The microspheres were coupled with gp120 protein using the xMAP antibody coupling kit as per the manufacturer. Coated microspheres were treated with heat inactivated, serially diluted mice sera for 2h at 37°C, washed, and then incubated with THP-1 cells. After overnight incubation of opsonized beads and cells, phagocytosis was measured by flow cytometry. ADCP scores were calculated as: (% microsphere (FITC) -positive cells × MFI of microspheres (FITC) -positive cells), where MFI is geometric mean fluorescence intensity. The ADCP score of titrated serum samples were plotted and used to calculate the area under the curves (AUC). To evaluate the ADCP capacity and account for Ig level differences among animal groups in each vaccination experiment, we calculated the AUC ratios of gp120-specific ADCP to gp120-binding total IgG. For example, we determined the ratio of the AUC of AA05-02 gp120-specific ADCP to AA05-02 gp120-binding total Ig. A similar analysis was performed for the REJO antigen.

### Statistical analysis

Statistical analyses were performed as indicated in the figure legends with t-test, Wilcoxon rank-sum test, or one or two-way ANOVA using GraphPad Prism 9.1.1 or R software.

## Results

### Altered antigenicity of SP-swapped Env

We investigated the effect of SP on the antigenicity and immunogenicity of Env from two clade B isolates: AA05 and AC02. The sequence of AA05 Env originated from an early-replicating (acute) isolate, whereas AC02 Env was derived from a late-stage (chronic phase) HIV-1 infection. Yolitz et al. (61) previously demonstrated, using the AA05 and AC02 gp120 proteins, that exchanging SPs between the two isolates influenced Env glycosylation and antigenicity as indicated by the reactivity with lectins (RCA, NPL, DC-SIGN) and select mAbs (2G12, 17b, and A32). In this study, we examined Env as soluble gp120 and membrane-bound gp160 proteins, and expanded the panel of mAbs beyond those tested previously (61), to further explore the influence of SP on CD4-binding site (CD4bs), V3, and the three types of V1V2 epitopes: conformational V2i, linear V2p, and quarternary V2q epitopes.

Antigenically, the WT AC02 gp120 exhibited stronger binding than WT AA05 with most tested mAbs, including V2i (830A, 2158, and 697), V3 (447), CD4bs (VRC01), and CD4i (17b) mAbs. However, both proteins showed similar reactivity with CD4bs mAb NIH45-46 and CD4i mAb A32 (**Figure 1**). The V2q mAb PG9, which preferentially binds to quaternary epitopes on the Env trimer (86–88), and the V2p mAb CH58 did not bind to AA05 or AC02 gp120, similar to the irrelevant control mAb 3685. In contrast, V3 mAbs 2219 reacted more strongly with AA05 WT than AC02 WT.

Expressing AA05 with AC02 SP (AA05-02, **Figure 1A**) increased AA05 binding by V2i (830A and 2158), V3 (2219), and CD4bs (VRC01) mAbs (**Figures 1B, C**). Hence, the mAb binding profile of AA05-02 more closely resembled that of the AC02 WT gp120 from which the SP was derived, despite having different gp120 encoding sequences (**Supplementary Figure S1**). The effects were specific as the binding of 697-D (V2i), 447 (V3), 17b (CD4i), and A32 (CD4i) mAbs was unaffected (**Figures 1B, C**). These data are in line with prior findings (55, 61), although the AC02 SP swap was seen to affect the reactivity of CD4i mAbs 17b and A32 (61).

The AA05 SP swap creating AC02-05 also caused changes in mAb reactivity (**Figure 2**). AC02 and AC02-05 had identical gp120 protein sequences and differed solely in their SPs (**Figure 2A**; **Supplementary Figure S1**), but AC02-05 exhibited reduced binding to most mAbs including V2i mAbs 830A, 2158, and 697D, relative to AC02 WT (**Figures 2B, C**). In contrast, AC02-05 had enhanced binding with V2p mAb CH58, suggesting the SP effect on the preponderance of distinct V1V2 conformations as detected by the V2i and V2p mAbs. The reactivity with V3 (447, 2219) and CD4i (A32) mAbs were unchanged. Overall, data in **Figures 1**, **2** demonstrated the capacity of SPs to modulate the antigenicity of gp120 proteins to reflect the characteristics of the parental proteins from which the SPs were derived.

**Figure 3:**
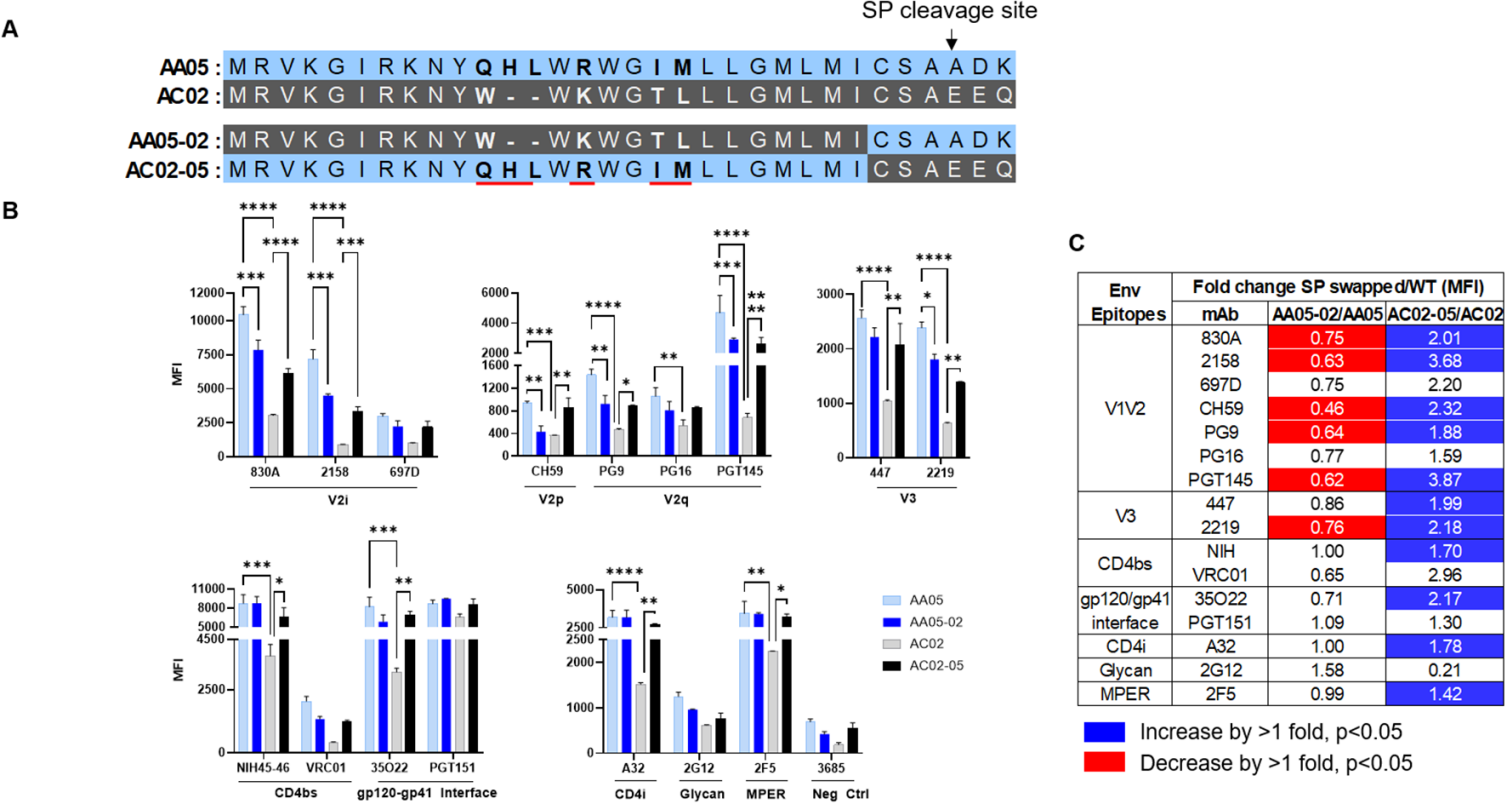
Changes in antigenicity of signal peptide (SP) DNA immunogens. **(A)** Schematic representation of WT and SP swapped gp160 Env. Dashes **(-)** indicate missing residues. Differences between the SP are shown in bold and are underlined. **(B)** Mouse muscle cell line C2C12 were transfected with gp160 expressing plasmids. Cells were probed with mAbs, 24 hours post-transfection followed by detecting the ligand binding by flow cytometry. *p < 0.05; **p < 0.01; ***p < 0.001; ****p < 0.0001 by 2-way ANOVA, p>0.05 was left unmarked. **(C)** Summary of the changes in antigenicity of gp160 proteins upon SP swapping. Fold changes (MFI) of SP swapped/WT were calculated for each mAb tested from **(B)**. Significant increases or decreases are marked with blue (fold change of >1 with p<0.05) or red (fold change of <1 with p<0.05), respectively.

We next tested the antigenicity of membrane-bound full-length gp160 proteins (**Figure 3**). A mouse myoblast cell line, C2C12, was tested to simulate the cell type that expresses the protein upon DNA vaccination. We transfected C2C12 cells with full-length WT and SP-swapped gp160 DNA and probed the membrane-bound Env by flow cytometry with mAbs targeting different Env regions. Overall the gp160 antigenicity profile differed from that of gp120 (**Figure 3B** vs. **Figures 1**, **2**). The profile was maintained when we normalized the Env expression over the respective MFI values of PGT151, a gp120-gp41 interface mAb which reacted with all four Env proteins to comparable extents (**Supplementary Figure 2**). Importantly, upon SP swapping, we observed alterations in mAb reactivity that reflected this pattern. Thus, recognition of AA05-02 by specific mAbs was diminished relative to the parental AA05, while the recognition of AC02-05 was enhanced (**Figures 3B, C**). This trend was consistent across mAbs targeting various Env regions, including V2i, V2p, V3, and select CD4bs epitopes. Among the V2q mAbs with a preference for quaternary epitopes on trimeric Env, PG9 and PGT145 also showed SP-dependent differences in binding: Envs with the AA05 SP exhibited stronger binding than Envs with the AC02 SP. Of the two gp120-gp41 interface mAbs tested, PGT151 showed similar rectivity with all four Envs, whereas 35O22 trended with higher binding to Envs with AA05 SP versus AC02 SP. Additionally, the CD4i (A32), V3 (447, 2219), and gp41 MPER (2F5) mAbs also demonstrated greater binding to the SP-swapped AC02-05 variant vs. AC02 WT. Taken together with data in **Figures 1**, **2**, these data demonstrated SP-induced alterations of Env antigenicity that varied significantly depending on the Env formats.

### Altered Env glycan composition by SP swapping

Altered binding of mannose specific mAb 2G12 to SP-swapped gp120 proteins vs their WT counterparts (**Figures 1**, **2**) indicated the influence of SP on Env glycosylation, as denoted in our past studies with virus-associated Env (45, 55). Thus, we assessed AA05 WT, AC02 WT and the respective SP-modified gp120 proteins for their glycosylation site occupancy and composition by mass spectrometry (MS) (79). The proteins were digested with different proteases to generate multiple peptides for each glycosite, which were then treated with Endo H and PNGase F enzymes to result in the introduction of novel masses that enabled the identification of peptides carrying high-mannose, complex-type, or unglycosylated forms. Since Endo H cleaves both high-mannose and hybrid-type glycans, the latter were grouped with high-mannose glycans for analysis purposes. The ratio of high-mannose to complex-type glycans was then calculated for each glycosite.

**Figure 4:**
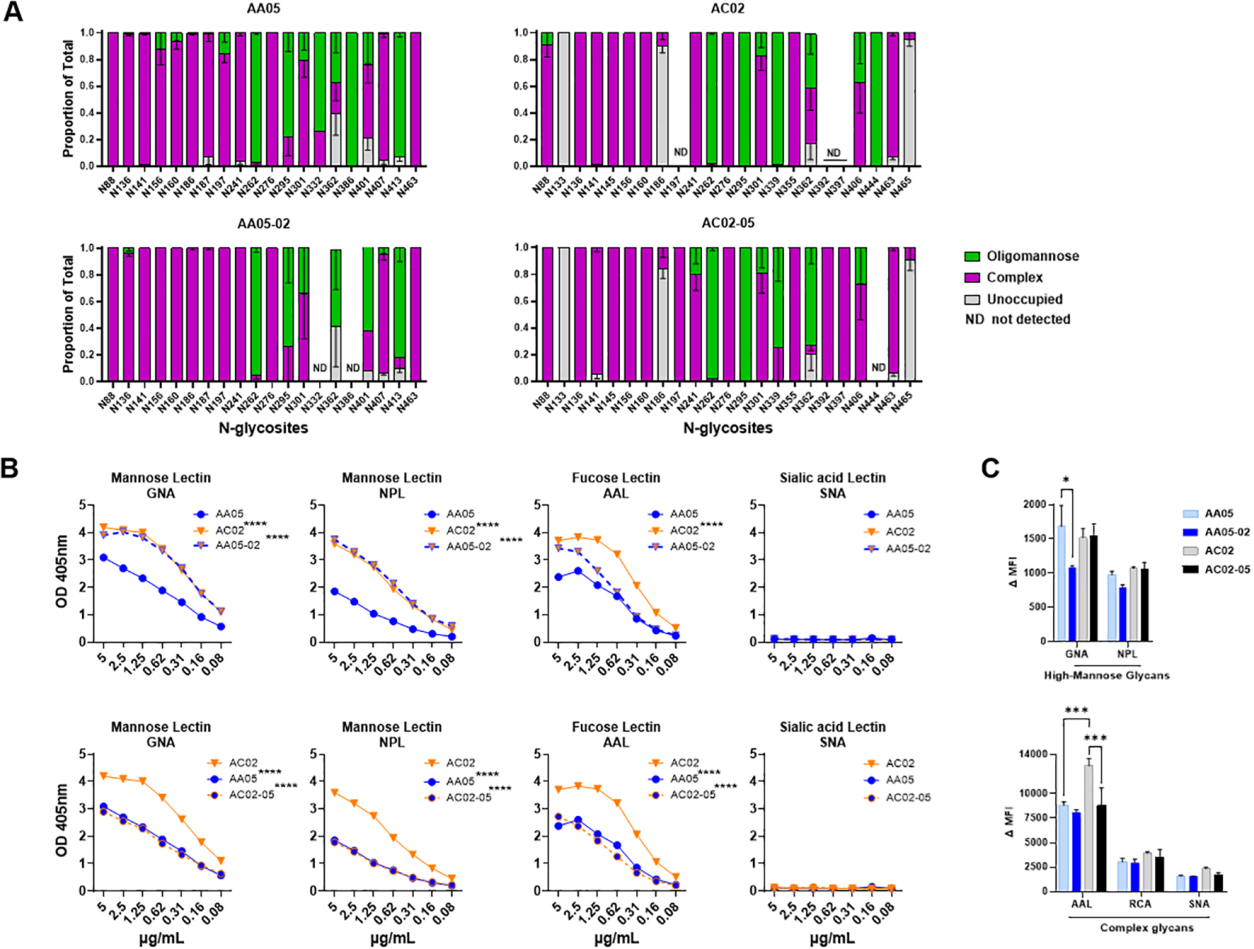
Changes in glycosylation of Env upon signal peptide (SP) swapping. **(A)** Relative amounts of unoccupied, complex and oligomannose/hybrid glycans at each glycosites on SP-swapped vs WT gp120 proteins as determined by LC-MS/MS. ND: glycosites not detected. **(B)** WT and SP swapped gp120 proteins were coated onto ELISA plates (2 μg/ml) and probed with serially diluted biotinylated lectins. Representative data are shown depicting the binding of biotinylated lectins specific to mannose, fucose, and sialic acid sugars on the N-linked Env glycans. **(C)** Mouse muscle cell line C2C12 were transfected with gp160 expressing plasmids. Cells were probed with lectins, 24 hours post-transfection followed by detecting the ligand binding by flow cytometry. Comparisons were made between AA05 versus AC02, AA05 versus AA05-02 and AC02 versus AC02-05. *p < 0.05; ***p < 0.001; ****p < 0.0001 by 2-way ANOVA, p>0.05 was left unmarked. nd, not detected.

The AA05 and AC02 gp120 proteins possess 24 and 27 potential N-linked glycosylation sites (PNGs), respectively (**Supplementary Figure S1**). The MS analyses were able to detect 20, 18, 20, and 22 glycosites for AA05 WT, AA05-02, AC02 WT and AC02-05, respectively (**Figure 4A**). Comparison of AA05 and its SP variant AA05-02 revealed glycan changes in the V1V2 (N156, N160), C2 (N197), V3 (N301), C3 (N362) and V4 (N401, N413) regions and no alterations at the N276 glycan near the CD4-binding site (15, 89) and at the conserved N262 glycan important for gp120 folding (90). Of note, the V1V2 (N156, N160) and C2 (N197) N-glycans on AA05 contained both high-mannose and complex-type sugars, whereas the AA05-02 variant exhibited exclusively complex sugars at these sites. In contrast, the N362 glycan, which is part of the 2G12 epitope, showed a shift in AA05-02 to solely high-mannose, as opposed to the mixed composition in AA05, corroborating the increased binding of mannose-specific 2G12 to AA05-02 versus AA05.

Changes in glycan composition were also observed between AC02 and its SP variant AC02-05. Of 19 detected glycosites on both proteins, glycan changes were noted at N88, N186, N241, N339, N362 and N406. No changes were detected at the V1V2 sites N156 and N160 where both proteins displayed complex-type glycans. Notably, AC02-05 had a higher proportion of high-mannose glycans on N362 compared to AC02 (**Figure 4A**). Despite this, AC02-05 showed reduced reactivity to mAb 2G12, indicating possible variations in the glycan structures important for 2G12 recognition that were not captured here. Indeed, the method used in this study provided a semi-quantitative evaluation of the broad glycan types at each site without identifying specific sugars that adorned each glycosite.

To detect finer changes in the sugar compositions, we probed the four gp120 proteins with lectins that recognize distinct sugar moieties: GNA (terminal α1–3 and α1–6 mannose on high-mannose glycans), NPL (α-linked mannose, preferring polymannose structures containing α-1,6 linkages), AAL (fucose on complex glycans) and SNA (sialic acid on complex glycans). The AC02 WT gp120 protein demonstrated a greater affinity for GNA, NPL, and AAL lectins compared to AA05 WT (**Figure 4B**) and neither protein showed any binding to the SNA lectin (**Figure 4B**). Similar to the mAb binding shown in **Figures 1**, **2**, the lectin binding to these gp120 proteins was also affected by SP swapping (**Figure 4B**). An increase in lectin binding was noted for AA05-02 over AA05 (top panels), while a decrease was observed for AC02-05 compared to AC02 (bottom panels). Notably, gp120s with same SPs (AA05-02 and AC02; AC02-05 and AA05) exhibited similar reactivities with GNA and NPL. This observation aligns with a prior study involving proteins from the same isolates, showing that SP exchanges conferred the glycan profile associated with the parental Env protein (61).

We also assessed the glycosylation changes for WT and SP-swapped gp160 proteins expressed on the surface of C2C12 cells using the lectin probes. All four Env proteins showed comparable NPL and RCA binding (**Figure 4C**). Little to no SNA binding was detected, similar to that seen with gp120 (**Figure 4B**). However, decreased GNA binding was observed with AA05-02 vs AA05 WT, while AAL binding to AC02-05 was lower than that to AC02 WT. These results were consistently observed after normalization of Env expression as measured with mAb PGT151 (**Supplementary Figure 2**). Thus, SP swapping altered lectin reactivity with gp160, albeit with a distinct pattern from that seen with gp120. Altogether, the MS and lectin binding data demonstrated that the use of a heterologous SP to express Env can impart substantial alterations to the Env glycan composition.

### Altered Env immunogenicity by SP swapping

We next evaluated the impact of SP exchanges on Env immunogenicity by immunizing mice with WT and SP-exchanged Env using three vaccine formats: 1) gp120 proteins, 2) gp120 DNA plus proteins, and 3) gp120 DNA plus gp160 DNA.

**Figure 5:**
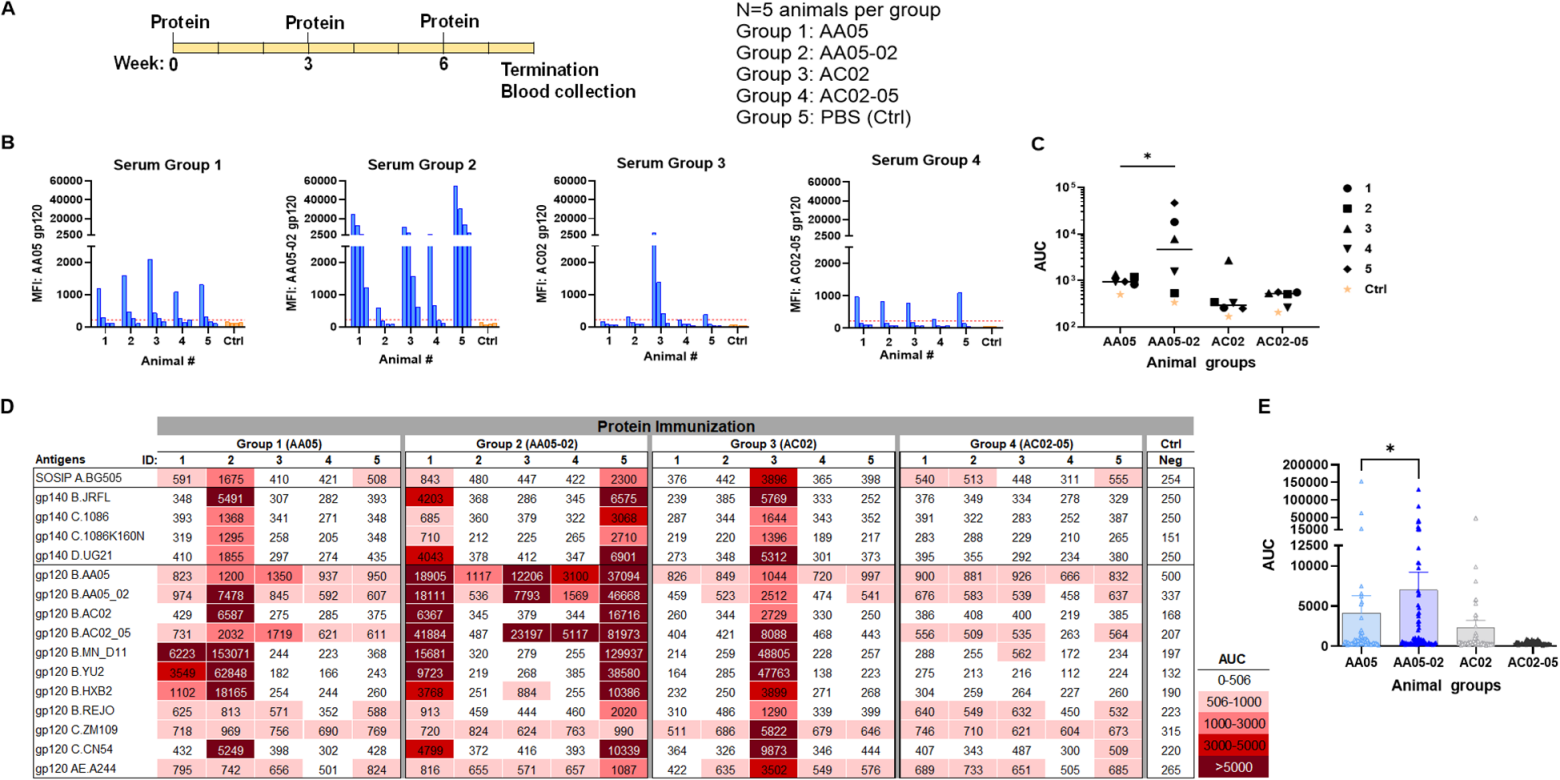
Antibody responses induced by vaccination with gp120 proteins. **(A)** Schematics showing the immunization schedule and the groups. BALB/c mice were immunized with gp120 proteins expressed with WT or swapped SP. Proteins were administered 3 times subcutaneously in the presence of adjuvant MPL/DDA. Control group received PBS and adjuvant (no gp120). **(B)** Sera from individual animals collected 2 weeks after the last immunization was tested with autologous proteins. Pooled sera from control group served as negative control. Gp120 proteins (2 µg/ml) were coated onto ELISA plates and reacted with serum IgG diluted four-fold starting at 1:100. Cut-off (224) shown in red dotted line is defined as the mean value of the MFI of Neg control at highest dilution tested plus 3SD value. **(C)** Area under titration curve (AUC) values were calculated from **(B)**. Horizontal line: median. *p = 0.028 by non-parametric Mann-Whitney test; p>0.05 was left unmarked. **(D)** Cross-reactivity of serum IgG from animals immunized with gp120 proteins. Plasma from individual mice were tested with SOSIP/gp140/gp120 proteins using multiplex Luminex assay. The serum IgG were diluted four-fold starting at 1:100. Area under titration curve (AUC) values were calculated and are colored as indicated. Cut-off (506) is defined as the mean value of the AUC of Neg control + 3SD value. **(E)** Scatter dot plot showing the comparison between the four animal groups from data in **(D)**. Mean ± SEM are shown. *p= 0.037, by non-parametric Mann-Whitney test, p>0.05 was left unmarked.

In the first experiment, we immunized five groups of female BALB/c mice (n = 5/group) and each group received three doses (3 ug/dose) of gp120 proteins with MPL-DDA adjuvant: AA05 WT (Group 1), AA05-02 (Group 2), AC02 WT (Group 3), AC02-05 (Group 4), and PBS control (Group 5) as outlined in **Figure 5A**. Two weeks after the final immunization, blood was collected for evaluation of the Ab responses.

We initially measured the levels of serum IgG to the autologous gp120 antigens (**Figures 5B, C**). Data from the Luminex multiplex bead assay showed that all mice in Group 1 generated low yet measurable Ab levels against the AA05 antigen, whereas only one of five animals in Group 3 showed an IgG response against the AC02 gp120 antigen. These findings suggest that both WT proteins had limited immunogenicity; however, AC02 WT was weaker than AA05 WT. In Group 4 (AC02-05), all mice also generated low or no autologous Ab responses. By contrast, 80% (4 out of 5) of animals in Group 2 (AA05-02) generated high levels of autologous Ab responses. Indeed, AA05-02 induced a stronger autologous binding Ab response compared to its WT counterpart AA05, (p=0.028, **Figure 5C**). The antigenic differences in gp120 proteins can result in qualitatively distinct antibody responses. However, the reasons behind the quantitative differences within the group remain unclear. One reason for the inconsistency could be that we tested the serum samples at a 1:100 dilution. Testing at a 1:50 dilution, especially for accommodating the group 3, might have reduced this inconsistency, although the low level of immune response would still have been evident.

To detect cross-reactive Ab responses, we measured serum IgG levels against a diverse array of Env antigens that included SOSIP, gp140, and gp120 from clades A, B, C, D, and CRF01_AE (**Figure 5D**). Animals in all four groups elicited Abs with varying degrees of cross-reactivity to the 16 tested antigens, although Group 4 showed relatively poor responses, consistent with the low or no autologous Abs detected in this group (**Figures 5B, C**). By comparing all data points from each group, we observed that Group 2 (AA05-02) had significantly stronger Ab reactivity with heterologous Env antigens than Group 1 (AA05 WT) (p = 0.037, non-parametric Mann-Whitney test; **Figure 5E**). In contrast, Group 4 (AC02-05) showed a trend of lower Ab reactivity than Group 3 (AC02 WT). These findings indicate that the AC02 SP swapping enhanced the immunogenicity of AA05 (AA05-02), while an opposite trend was exerted by the AA05 SP.

**Figure 6:**
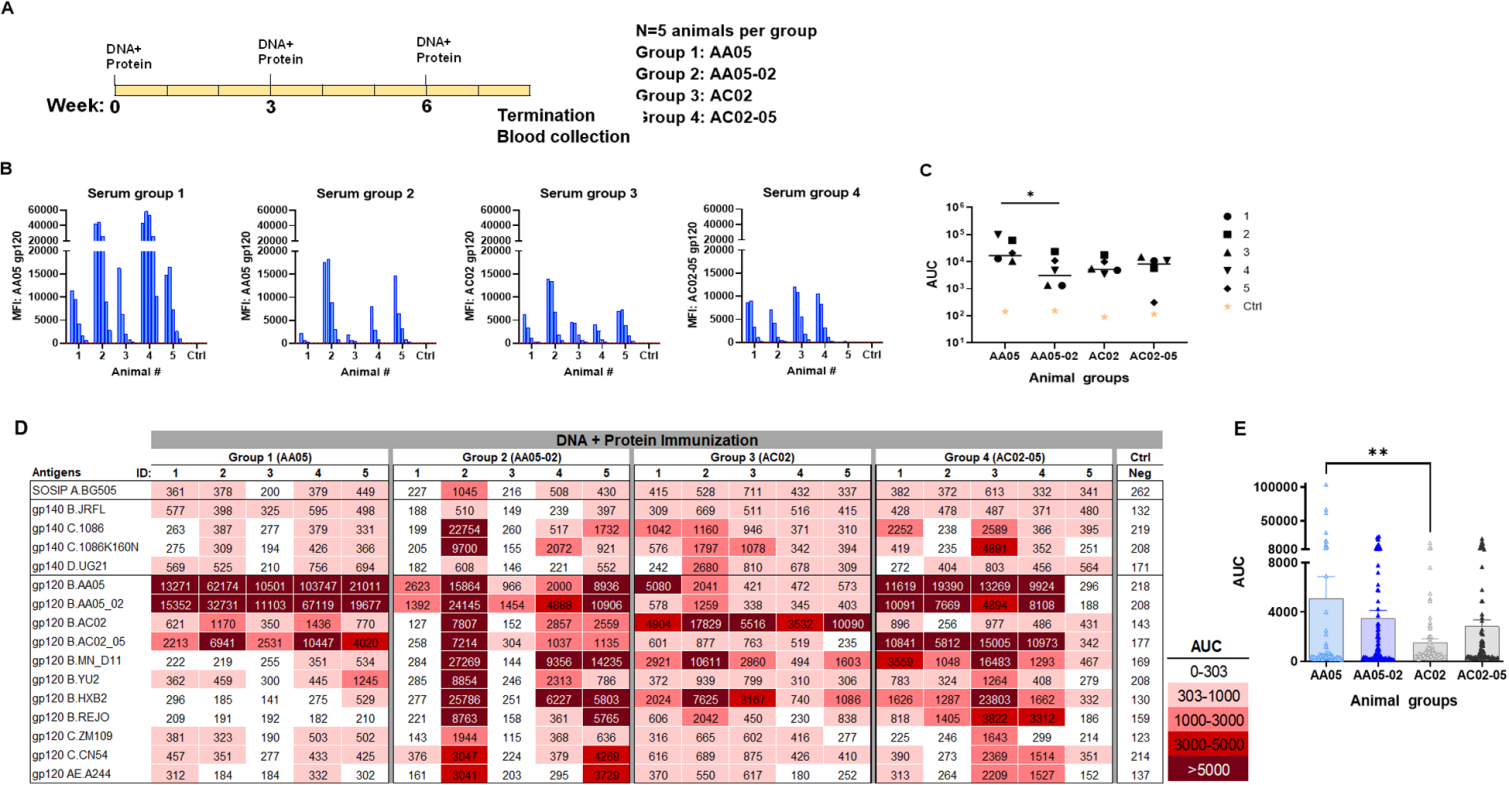
Antibody responses induced by vaccination with gp120 DNA and protein co-immunization. **(A)** BALB/c mice were co-immunized with gp120 DNA and gp120 protein (WT and swapped SP). **(B)** Serum from individual animals collected 2 weeks after the last immunization was tested with autologous proteins using multiplex Luminex assay. The serum IgG were diluted four-fold starting at 1:100. Pooled serum from unimmunized normal mouse (NMS) served as negative control (Ctrl). Geometric mean fluorescence intensity (MFI) are shown. Cut-off (80.8) shown in red dotted line is defined as the mean value of the MFI of Neg control at highest dilution tested plus 3SD value. **(C)** Area under titration curve (AUC) values were calculated from **Figure 7B**. Horizontal line: median; *, p= 0.047, by non-parametric Mann-Whitney test; p>0.05 was left unmarked. **(D)** Cross-reactivity of serum IgG from animals co-immunized with gp120 DNA and proteins. Sera from individual mice were tested with SOSIP/gp140/gp120 proteins using multiplex Luminex assay. The serum IgG were diluted four-fold starting at 1:100. Area under titration curve (AUC) values were calculated and are colored as indicated. Cut-off value (303) is defined as the mean value of the AUC of Neg control + 3SD value. **(E)** The AUC values for each group from **(D)** were plotted as scatter-plots. Mean + SEM are shown. **p= 0.005, by non-parametric Mann-Whitney test; p>0.05 was left unmarked.

In the second experiment, the immunogenicity of WT and SP-exchanged antigens was assessed by immunizing mice with a combination of gp120 DNA and gp120 proteins as shown in **Figure 6A**. Co-immunization with gp120 DNA and proteins elicited a more robust immune response than protein immunization alone (**Figure 6** vs **Figure 5**), similar to previous reports (91–95). All groups produced detectable Ab responses against their respective autologous proteins (**Figure 6B**). However, Group 1 (AA05) showed a greater response than Group 2 (AA05-02), while the responses of Groups 3 and 4 were comparable (**Figures 6B, C**). The binding responses to heterologous antigens followed a similar pattern, with serum IgG in Group 1 (AA05) trending to show higher binding than Group 2 (AA05-02), and Group 3 (AC02) displaying weaker responses than Group 4 (AC02-05) (**Figures 6D, E**). Group 1 (AA05) also had greater heterologous Ab responses than Group 3 (AC02). Comparison between Experiments 1 and 2 demonstrated the effects of SP swapping that differed depending on the vaccine formats.

**Figure 7:**
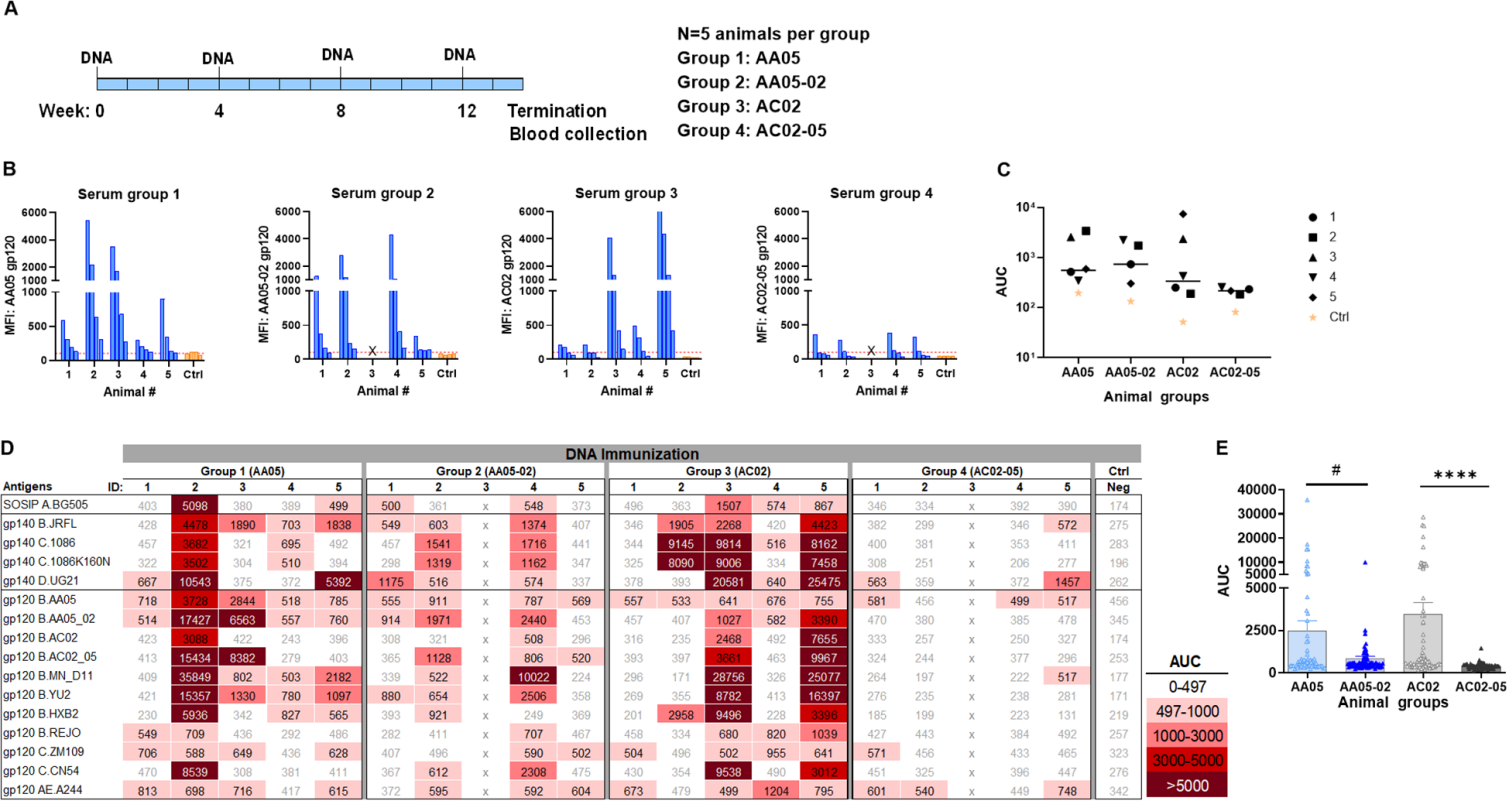
Antibody responses induced by vaccination with gp120 and gp160 DNA. **(A)** BALB/c mice were immunized with gp120 and gp160 DNA (1:1) (WT and swapped SP). **(B)** Serum from individual animals collected 2 weeks after the last immunization was tested with autologous proteins using multiplex Luminex assay. The serum IgG were diluted four-fold starting at 1:100. Pooled serum from unimmunized normal mouse (NMS) served as negative control (Ctrl). Geometric mean fluorescence intensity (MFI) are shown. Cut-off (167.8) shown in red dotted line is defined as the mean value of the MFI of Neg control at highest dilution tested plus 3SD value. **(C)** Area under titration curve (AUC) values were calculated from **Figure 9B**. Horizontal line: median; p>0.05 was left unmarked. **(D)** Cross-reactivity of serum IgG from animals immunized with gp120 and gp160 DNA. Sera from individual mice were tested with SOSIP/gp140/gp120 proteins using multiplex Luminex assay. The serum IgG were diluted four-fold starting at 1:100. Area under titration curve (AUC) values were calculated and are colored as indicated. Cut-off value (497) is defined as the mean value of the AUC of Neg control + 3SD value. **(E)** The AUC values for each group from **(D)** were plotted as scatter-plots. Mean + SEM are shown. ^#^p= 0.06, ****p <0.0001 by non-parametric Mann-Whitney test, p>0.05 was left unmarked. x, mice died.

In the third experiment, we evaluated the effect of SP exchanges on Ab responses elicited by a combination of gp120 DNA and gp160 DNA vaccines (1:1 ratio). The full-length gp160 DNA vaccine was included in order to elicit Ab responses against the membrane-bound native Env conformations present on virions and infected cells. Four groups of mice were immunized with WT and SP-swapped variants as outlined in **Figure 7A**. All four groups developed Ab responses to the corresponding autologous proteins detectable above the background level (**Figures 7B, C**). The magnitudes of the autologous responses were comparable, although Group 4 (AC02-05) exhibited the lowest levels akin to those seen with gp120 protein immunization (**Figure 5**). Nonetheless, the autologous Ab responses elicited by the gp120 DNA+gp160 DNA vaccines were weaker than those elicited by gp120 protein only (**Figure 5**) and by the gp120 DNA+gp120 protein combination (**Figure 6**).

Immune sera from the third experiment were also assessed for cross-reactive binding to the same panel of antigens. Although the autologous Ab responses were relatively weak, all four groups had varying levels of serum IgG against multiple heterologous antigens (**Figures 7D, E**). The collective data in **Figure 7E** showed that the two groups receiving the WT immunogens (Group 1: AA05 and Group 3: AC02) had higher levels of cross-reactive Ab responses than the groups immunized with the respective SP-swapped variants (**Figures 7D, E**). These data provided more evidence for the role of SP in influencing Env immunogenicity and confirmed the differential effects of SP swapping on the distinct Env vaccines.

### Epitope specificities of Abs induced by WT and SP-swapped Env vaccines

**Figure 8:**
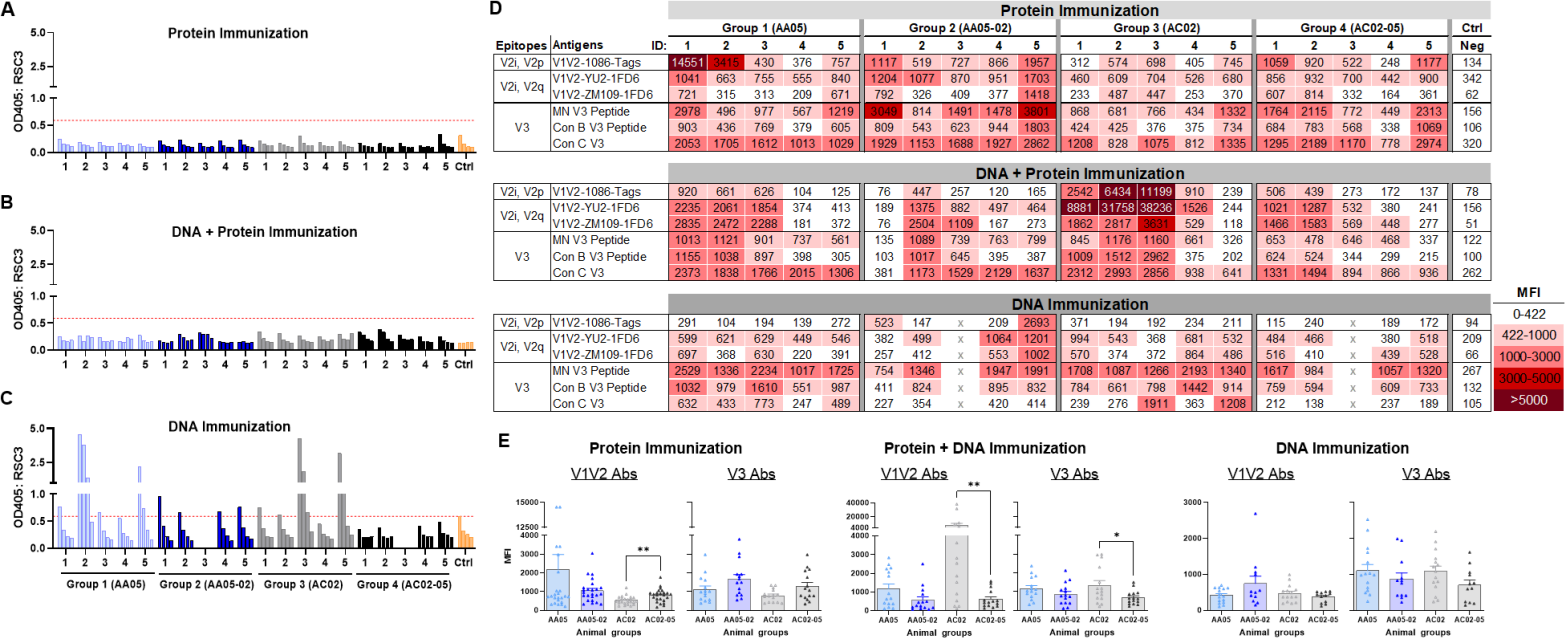
Binding of serum IgG to epitope specific antigens. Serum IgG from animals immunized with WT and SP swapped antigens were tested for their binding to different antigens representing the gp120 core, V1V2 (V2i/V2p/V2q) and V3 epitopes. Binding of serum IgG from **(A)** Protein, **(B)** DNA and Protein, and **(C)** DNA immunizations to core antigen RSC3 by ELISA. Serially diluted sera (4-fold) starting from 1:100 were reacted to RSC3 antigen (1µg/ml) coated onto the ELISA plates. Pooled serum from unimmunized mouse served as negative control (Ctrl). The absorbance values (OD450 nm) of sera are plotted and shown. Red dotted line denotes the OD405 of control sample at 1:100 dilution. **(D)** Binding of serum IgG to different scaffold/peptide antigens representing V2i, V2p, V2q and V3 epitopes using multiplex Luminex assay. Sera diluted at 1:100 dilution were tested and are colored as indicated. Mean fluorescence intensity (MFI) are shown. Cut-off value (422) is defined as the MFI of the Neg control + 3SD value. **(E)** The MFI values for each animal and immunization group from **(D)** were plotted as scatter-plots. Mean + SEM are shown. p values as measured by non-parametric Mann-Whitney test, *, p < 0.05; **, p < 0.01. p>0.05 was left unmarked. x, mice died.

To identify the specific Env regions targeted by the vaccine-elicited Abs, we examined serum IgG reactivity with a panel of antigens representing the CD4-binding site (CD4bs), V1V2, and V3 (**Figure 8**). To detect Abs against the CD4bs, we used the RSC3 antigen presenting the antigenic structure of the CD4 binding site (CD4bs) and lacking the V1V2 and V3 regions of the Env (96). All mice immunized with gp120 protein and gp120 DNA+protein vaccines had no serum IgG reactivity with RSC3 (**Figures 8A, B**). In contrast, a fraction of animals immunized with gp120 DNA+gp160 DNA vaccines generated high levels of Abs against RCS3: two animals in Group 1 (AA05 WT) and two animals in Group 3 (AC02 WT) (**Figure 8C**). Animals in Groups 2 and 4 that were immunized with SP-swapped Env had poor or no RCS3-binding Ab responses (**Figures 8A–C**).

To evaluate Ab responses targeting V1V2, we performed the Luminex multiplex bead assay utilizing antigens bearing different types of V1V2 epitopes: C.1086 V1V2-tags presenting V2i and V2p epitopes, B.YU2 V1V2-1FD6 and C.ZM109 V1V2-1FD6 constrained to display V2i and V2q epitopes (63, 86, 95, 97, 98). We also included cyclic V3 peptides of B.MN and the consensus B and C sequences to detect Ab responses to the V3. The data revealed that all groups of animals immunized in the three experiments generated serum Abs capable of binding to multiple V1V2 and V3 antigens to varying levels above background (**Figure 8D**). Among gp120 protein vaccines, AC02-05 induced higher Ab responses to V1V2 and V3 as compared with AC02 WT (**Figure 8E** left panel). In contrast, DNA+protein coimmunization AC02 WT resulted in significantly higher levels of Abs against V1V2 and V3 as compared to AC05-02. Immunization with AA05 WT trended to elicit higher V1V2 and V3 Abs compared to AA05-02 however it did not reach statistical significance (**Figure 8E** middle panel). In contrast, the four groups immunized with gp120 DNA+gp160 DNA vaccines did not show discernible differences (**Figure 8E** right panel).

### ADCP activities induced by WT and SP-swapped Env vaccines

**Figure 9:**
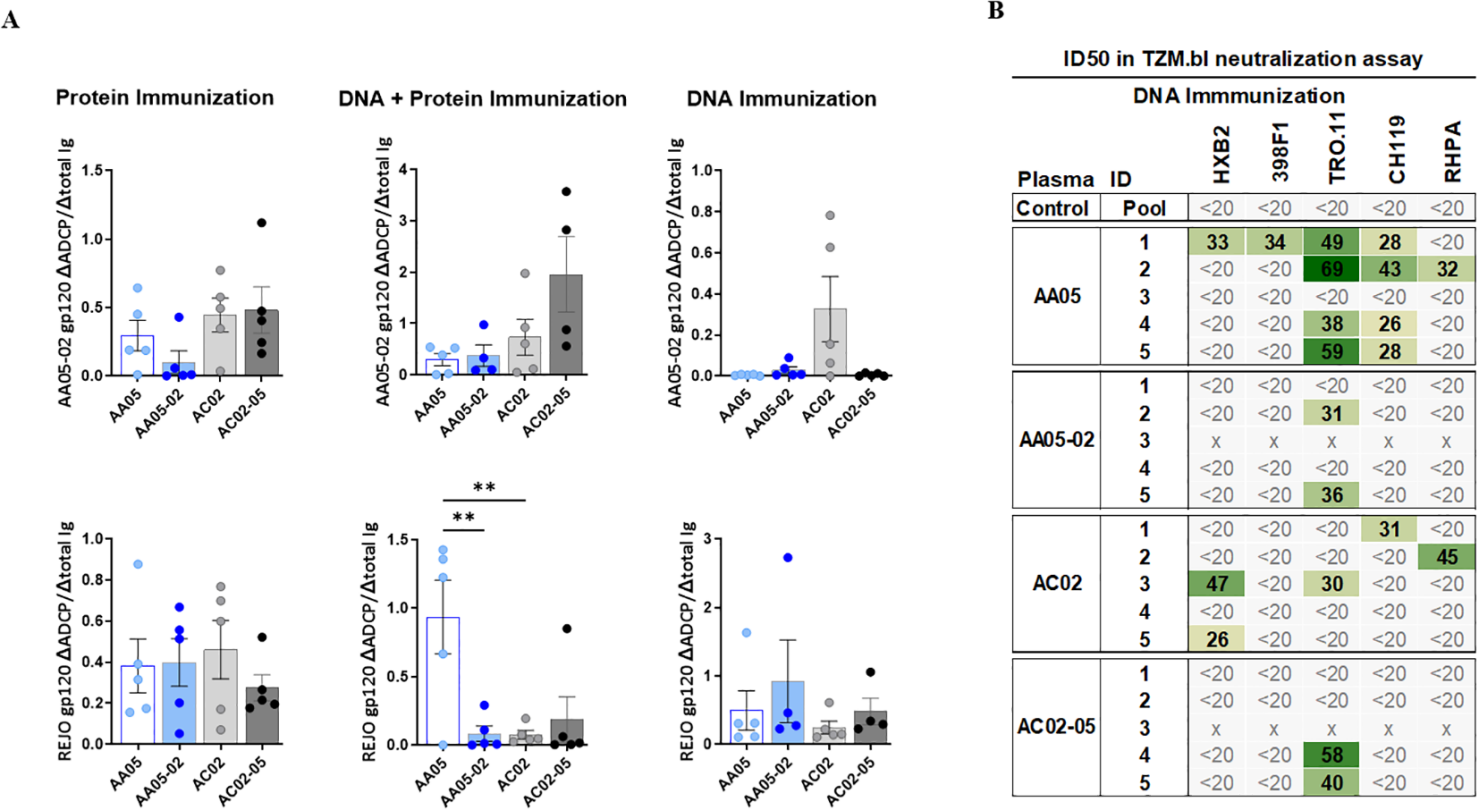
Vaccine-Induced antibodies mediate antibody-dependent cellular phagocytosis (ADCP) and virus neutralization. Serially diluted samples from protein, DNA+ Protein, and DNA immunizations were tested for **(A)** ADCP using phagocytic THP-1 cells and fluorescent beads coated with AA05_02 (upper panel) and REJO gp120 proteins (lower panel). Pooled sera from unimmunized animals was tested in parallel as negative control. Titration curves were plotted for ADCP scores and area under titration curve (AUC) were calculated. AUC values for negative controls were subtracted for both ADCP and IgG binding. ADCP potency of each sera was calculated as the ratio of ΔADCP/ΔIgG against AA05_02 and REJO gp120 and shown. **(B)** Serum samples collected from mice immunized with gp120 and gp160 DNA immunogens were tested for neutralization of Tier 1 (pseudovirus HXB2) and Tier 2 viruses (pseudovirus 398F1, TRO.11, CH119 and RHPA infectious virus) in a standard assay using the TZM.bl target cells. Pooled serum from unimmunized normal mouse served as negative control (Ctrl). Mouse sera neutralization titers are reported as serum dilution required to inhibit 50% of virus infection (ID50). Reciprocal serum ID50 values that can be measured above the cut-off (1:20) are indicated in graded green. x, animal died. **p < 0.01; p>0.05 was left unmarked by ANOVA.

To determine the impact of SP swapping on the generation of functional Abs, we first evaluated the capacity of vaccine-induced serum Abs to mediate antibody-dependent cellular phagocytosis (ADCP) in the THP-1 ADCP assay (99, 100). Serially diluted sera were incubated with AA05-02 or REJO gp120 proteins coated on fluorescent beads and then added to the THP-1 phagocytic cells (**Figure 9A**, **Supplementary Figure 3**). Results showed that sera from all vaccinated groups elicited Abs that exhibited ADCP capabilities against the gp120 proteins, with no discernible differences between animals that received either the wild-type or SP-altered immunogens (**Supplementary Figure 3**). To compare the potency of Abs elicited by WT versus SP-swapped Env in groups of animals in each vaccination experiment, while taking into account the varying Ab levels in individual animals, we calculated the ratios of ADCP over IgG levels against the respective gp120 antigens (**Figure 9A**). Highly variable ADCP potencies were observed across groups in the three experiments. Among the three immunization experiments, only animals co-immunized with AA05 WT gp120 DNA + protein exhibited higher REJO gp120-specific ADCP potency compared to AA05-02, suggesting a qualitative difference in the gp120-specific Abs elicited by the WT AA05 compared to its SP-swapped variant in this immunization setting.

### Virus neutralizing activities induced by WT and SP-swapped Env vaccines

To determine the impact of SP swapping on the capacity of Env vaccines to generate neutralizing Abs, sera from animals immunized in the three experiments were tested against one tier 1 and four tier 2 HIV-1 strains in the TZM.bl neutralization assay (101). No virus neutralization was detected in sera from mice immunized with WT or SP-swapped gp120 proteins (first experiment) and from mice co-immunized with the corresponding gp120 DNA+protein vaccines (second experiment) (**Supplementary Figure 3D**). However, a fraction of animals in each of the groups that received gp120+gp160 DNA vaccines (third experiment) exhibited neutralization against at least one of the viruses tested, with ID_50_ titers ranging from 26 to 69 (**Figure 9B**). Notably, four out of five animals in the AA05 WT group displayed neutralization against one or more viruses (IC_50_ of 26-69) in comparison to only two animals in the AA05-02 group (IC_50_ of 31-36) (**Figure 9B**). Neutralization breadth also differed: 44% for AA05 WT vs 8% for AA05-02. In the AC02 group four out of five animals showed neutralization against one or two viruses (IC_50_ of 26-47; neutralization breadth 20%) in comparison to only two animals in the AC02-05 group (IC_50_ of 40-58; neutralization breadth 8%). The neutralization titers elicited by AA05 and AC02 WT DNA vaccine were marginal, but these neutralizing responses were diminished upon substituting their SPs.

## Discussion

This study investigated the impact of SP on the antigenic and immunogenic properties of HIV-1 Env. Using two clade B Env immunogens, AA05 and AC02 from acute and chronic isolates, respectively, and a panel of mAbs against different sites of Env, we demonstrated that the antigenic qualities of these Env immunogens were interchangeable by swapping their SPs. This modulation was apparent but distinct for soluble gp120 versus membrane-bound gp160 (**Supplementary Figure 4**). Moreover, SP exchanges altered mAb reactivity against disparate Env regions, including V1V2, V3 crown, and CD4bs, suggesting widespread effects on Env conformation. Importantly, AA05 and AC02 Env also showed differential immunogenicity that was consistently observed in the three vaccine formats, and SP-swapping led to specific alterations in the levels, breadth, and functions of the elicited antibody responses (**Supplementary Figure 4**). Collectively, the study highlights the modulatory potential of SPs on Env antigenicity and immunogenicity.

One notable effect of the SP swapping was observed on Env recognition by V1V2-specific mAbs. The effects on the V2i epitope recognition as detected by mAbs 830A, 2158, and 697D were particularly pronounced. Notably, alterations in V2i mAb reactivity with swapped versus the respective WT gp120 proteins, e.g. AA05 versus AA05-02, occurred despite their identical gp120 residues, suggesting that the SP swapping shapes the structural preference and/or flexibility of the V1V2 domain independent of the gp120 primary sequences. The V2i mAbs, such as 830A and 697D, recognize the V2C strand of V1V2 region in a β-sheet conformation, albeit from different angles (31, 32, 70). The flexible V2C strand can also adopt alpha helical structures as recognized by the V2p mAbs (63). Lower binding of AA05 and AC02-05 gp120 proteins, as compared to AC02 and AA05-02, by all three V2i mAbs suggests that the AA05 SP biases the V2C structure away from the β-sheet conformation towards an α-helix-coil structure. In support of this, AC02-05 also showed increased binding to the V2p mAb CH58. In the case of 697-D, its V2i epitope encompasses key contacts involving F176, Y177, D180, Y191, and L193, and mutations affecting F176 disrupted the mAb binding (31). The presence of L176 in AA05 likely explains the lack of reactivity of 697D to AA05, which could not be rescued in the SP-swapped AA05-02.

Comparison of AA05 and AC02 WT gp120 proteins further revealed that AC02 had superior antigenicity but weaker immunogenicity. Upon SP swapping, AA05-02 gp120 displayed increased antigenicity and immunogenicity over AA05 WT, resulting in the production of Abs with robust levels of cross-reactive binding. In contrast, AC02-05 protein had reduced antigenicity and lower immunogenicity compared to AC02 WT (**Supplementary Figure 4**). These data provide evidence for the role of SPs in regulating gp120 recognition by antibodies and more importantly the ability to modulate antibody responses upon immunization.

The ability of SP to influence Env antigenicity was also observed in the context of membrane-bound gp160, although an opposing pattern emerged. Unlike the results seen with gp120 protein, the AA05-02 gp160 protein expressed on the surface of mouse muscle cell line C2C12 showed lower reactivity with V1V2 mAbs than the AA05 WT counterpart and increased V1V2 mAb reactivity was seen with AC02-05 versus AC02 WT. The differing steric constraints and V1V2 epitope presentations between monomeric and trimeric Env forms (102–105) may contribute to the discrepancies. Nevertheless, the altered antigenicity by the SP used for Env expression remained notable for both soluble gp120 and membrane-bound gp160 and could have implications on the immunogenicity of different Env vaccines.

Co-immunization with gp120 protein and gp120 DNA vaccines pointed to a negative effect of SP swapping. This was most apparent from the reduced Ab responses elicited by AA05-02 as compared to the AA05 WT counterpart. A similar trend was also noted upon immunization with the gp120+gp160 DNA vaccines. Notably, the WT gp120+gp160 DNA vaccines of AA05 and AC02 elicited Abs reactive with the core RSC3 antigen, indicative of the elicitation of CD4bs-type Abs. Furthermore, the AA05-WT DNA vaccines induced Abs with low level cross-clade neutralization capabilities, which were not detected after immunization with the AA05-02 vaccines. It is important to note that the boost schedule in experiment 3 was four weeks apart, compared to three weeks in experiments 1 and 2. This timing can impact both the immunogenicity and the functions of the antibodies produced, with longer intervals often being more effective. However, it is not certain that this delayed schedule in experiment 3 led to the development of neutralizing antibodies, and this could be explored further in future studies. Another aspect not explored in this study, but which could influence immunogenicity, is the T cell immune response. Understanding how T cell responses to the different immunogens (WT and SP-swapped) might have impacted the results would be valuable for future research. Due to the limited availability of serum samples, it was also not possible to define the nature and specificity of the observed neutralizing responses in these animals, one of the limitations of this study. Eliciting Abs capable of neutralizing a broader range of tier 2 isolates has proven extremely difficult to achieve by vaccination. Preclinical studies using soluble forms of the Env trimer stabilized in a configuration similar to that presented on native viral particles (SOSIP and its variants) have reported induction of robust autologous but not heterologous neutralizing Abs (35, 106–111). Immunization of rabbits with a regimen of vaccinia virus prime-gp120 protein boost elicited Abs capable of neutralizing select heterologous tier 2 isolates albeit at low titers (reciprocal ID_50_ ranging from 20 to 100) (112, 113). Another study investigating how vaccination with immune complexes (ICs) may impact the elicitation of functional antibodies also reported low and limited tier 1 and tier 2 neutralizing activity in rabbits (ID_50_: 32 to 335) (98). Immunization of macaques with a messenger RNA (mRNA) vaccine co-expressing membrane-anchored Env and simian immunodeficiency virus (SIV) Gag proteins to generate virus-like particles (VLPs) successfully induced Abs capable to neutralize a limited number of heterologous tier 2 viruses but also at low potencies (114). Despite numerous attempts, the generation of high titers of Abs capable of neutralizing a broad array of heterologous tier 2 viruses by vaccination has not yet been achieved. The neutralization potency of serum Abs induced in mice immunized in this study was also low, with ID_50_ titers of 26-69. Nonetheless, the findings implicate the benefit of incorporating full-length Env with the native SP for an Env immunogen construct. Additionally, the study provided examples (AA05 vs. AC02) that not all Envs are equal and that the Env strain selection is one of the critical parameters for developing Env-based vaccines, although the desirable Env properties remain unclear. The SPs of AA05 and AC02 exhibit six amino acid differences at positions 11, 12, 13, 15 in the N-region and 18, and 19 in the H-region. We have previously shown that mutating the residues at position 12 and 15 can alter the sensitivity of mutant viruses (REJO and JRFL) to neutralization by V1V2 and V3 mAbs (45). In this study, antigenic changes in WT (AA05, AC02) and SP swapped (AA05-02, AC05-05) Env were also more pronounced with V1V2 and V3 mAbs. However, the specific roles and contributions of individual SP residues in shaping the Env antigenic profile require further research. At position 12 in the N-region, the AA05 SP includes a histidine (H12), which is absent in the AC02 SP. Loss of H12 in the Env SP has been associated with the transition from acute to chronic viruses (47, 48). A similar signature motif was reported in the Env SP of SIV (49). A study by da Silva et al. revealed loss of neutral and basic amino acids at the N-terminus of the Env SP during virus evolution from early/acute to late/chronic stages (115). SP sequence is also functionally connected to regulating levels of Env expression at different stages of infection (47). The Env SP uniquely has a higher number of positively charged residues compared to the surface proteins of other viruses e.g., influenza HA and SARS-CoV2 spike. In total, the AA05 SP has five basic residues in the SP N-region, compared to four in the AC02 SP. The positive charges in the SP N-region was demonstrated to be important in maintaining efficient translocation in the ER and post-translational modifications including glycosylation (56, 116, 117). Indeed, lectin binding shows differences in fine glycan specificities in the SP swapped versus WT proteins. These data advocate important roles of SP that are beyond ER targeting. SPs are more intricately involved in protein characteristics and functions than noted before. The SP has been implicated in governing HIV-1 Env glycosylation (33). Our published data demonstrates that swapping the SP from another HIV-1 strain or even a single mutation in the SP can alter the relative proportion of high-mannose, and complex glycans on N-glycosites on Env (45, 55). In this study, gp120 proteins expressed with the AC02 SP (AA05-02 and AC02) had higher levels of high-mannose and fucose-bearing complex glycans compared to those with the AA05 SP (AA05 and AC02-05). Correspondingly, AA05-02 and AC02 immunized animals elicited stronger binding antibody responses than AA05 and AC02-05. When DNA was included in the regimen (Exp 2 and 3), a similar association was observed: AA05 was more reactive to high-mannose specific lectins and induced a stronger binding antibody response than AA05-02. However, in case of AC02, no discernible association between glycosylation level and antibody response could be observed based on the binding of high-mannose specific lectins. Nonetheless, AC02 gp160 Env showed greater binding to AAL compared to AC02-05, which was reflected in the antibody response, where AC02 induced a stronger immune response compared to AC02-05. Determining the optimal glycan pattern for immunogenicity requires further investigation. Current immunization schema followed in the field use immunogens as DNA, mRNA and protein in various vaccination combinations however, in most cases combinations of DNA and protein or mRNA and protein is preferable due to their ability of induce an Ab response of higher magnitude compared to either alone. Thus, it is important to consider the antigenicity and glycosylation profile of the Env that will be produced via DNA and mRNA immunogens. Based on our results, using the DNA and protein combination approach for immunization is best suited to elicit high titer binding Abs, and inclusion of native SP is preferable.

Overall, this study provides the first evidence demonstrating that the use of heterologous SPs to express Env immunogens was accompanied by significant immunogenic changes. Notably, these changes impact the induction of cross-reactive binding and functional Abs. However, to better understand the implications and mechanisms underlying these observations, additional investigations are needed. Future studies should delve into the SP-mediated immunogenic shifts, the interplay between SPs and Env structures, and the relevance of SP in the broader context of Env vaccine design and efficacy.

## Data Availability

The original contributions presented in the study are included in the article/**Supplementary Material**. Further inquiries can be directed to the corresponding author.
