# Supplementary material for: Signal peptide exchange alters HIV-1 envelope antigenicity and immunogenicity
Source: Front Immunol. 2024 Sep 24;15:1476924. doi: 10.3389/fimmu.2024.1476924 (PMC11458420; doi:10.3389/fimmu.2024.1476924)
Supplement: Supplementary file 1 [file DataSheet1.pdf]

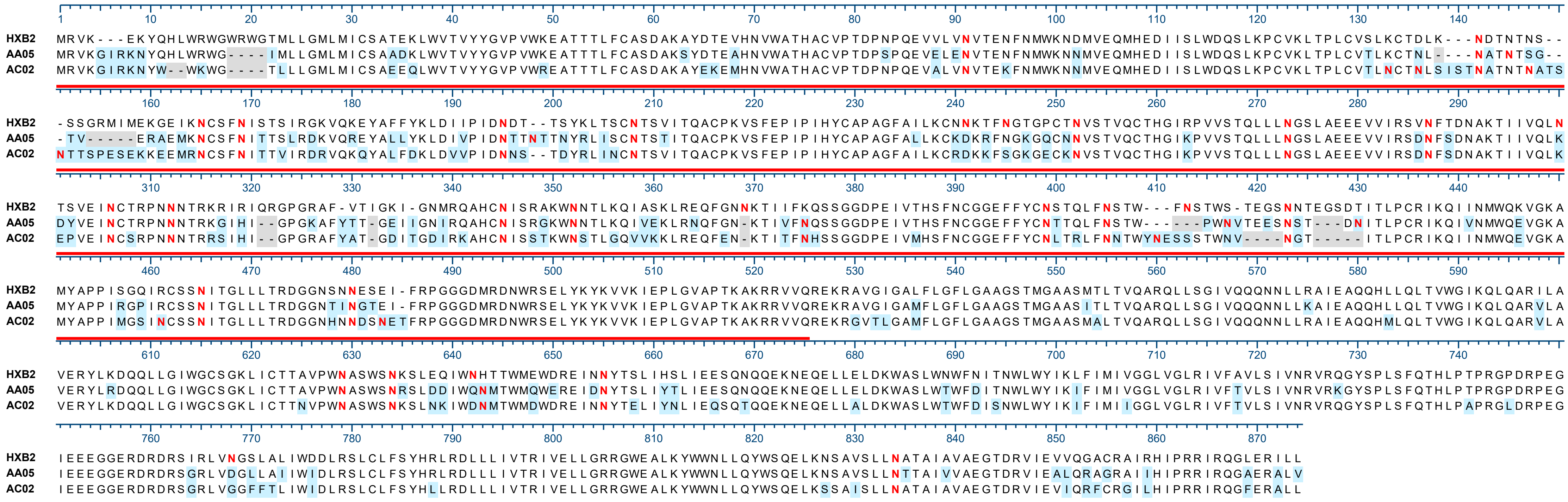

Supplementary Figure 1. Alignment of AA05 and AC02 amino acid sequence with HXB2 used as reference. The gp160 sequences were aligned using Lasergene 17.4.2. The sequences used to express recombinant gp120 protein is denoted by red line. The differences are shown in light blue background, missing residues are shown as dash and gray background. Potential N-linked glycosylation sites (PNGS) are highlighted in red and bold. N-Glycosite tool on Los Alamos website was used to find the PNGS.

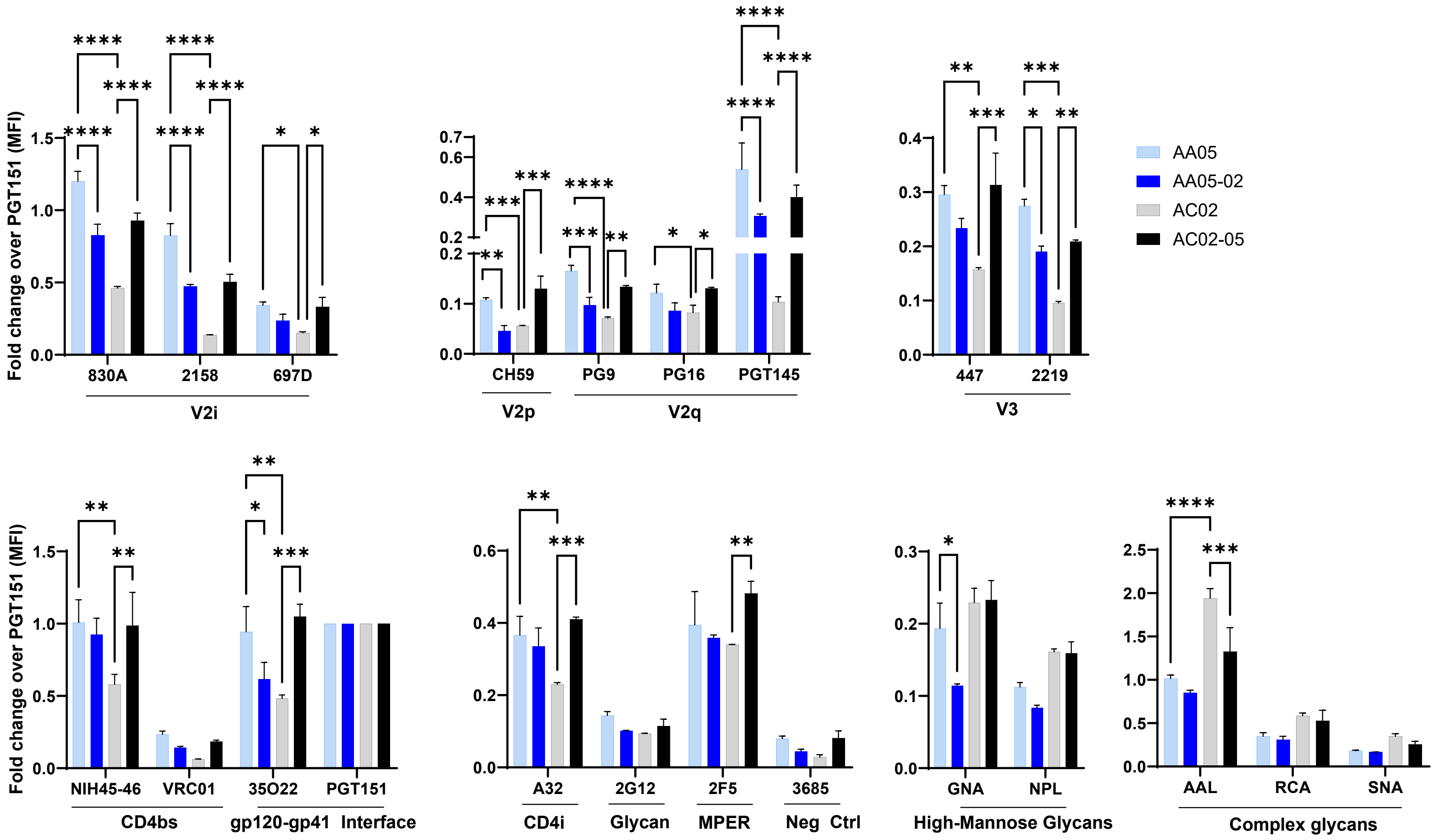

Supplemental Figure 2. Fold change in antigenicity and glycosylation of SP swapped DNA immunogens. Mouse muscle cell line C2C12 were transfected with gp160 expressing plasmids. Cells were probed with mAbs and lectins, 24 hours post-transfection followed by detecting the ligand binding by flow cytometry. Data from figures 3B and 4C were used to calculate the fold change in antigenicity and lectin binding over PGT151 mAb.*, $p < 0.05$; **, $p < 0.01$; ***, $p < 0.001$; ****, $p < 0.0001$ by 2-way ANOVA, $p>0.05$ was left unmarked.

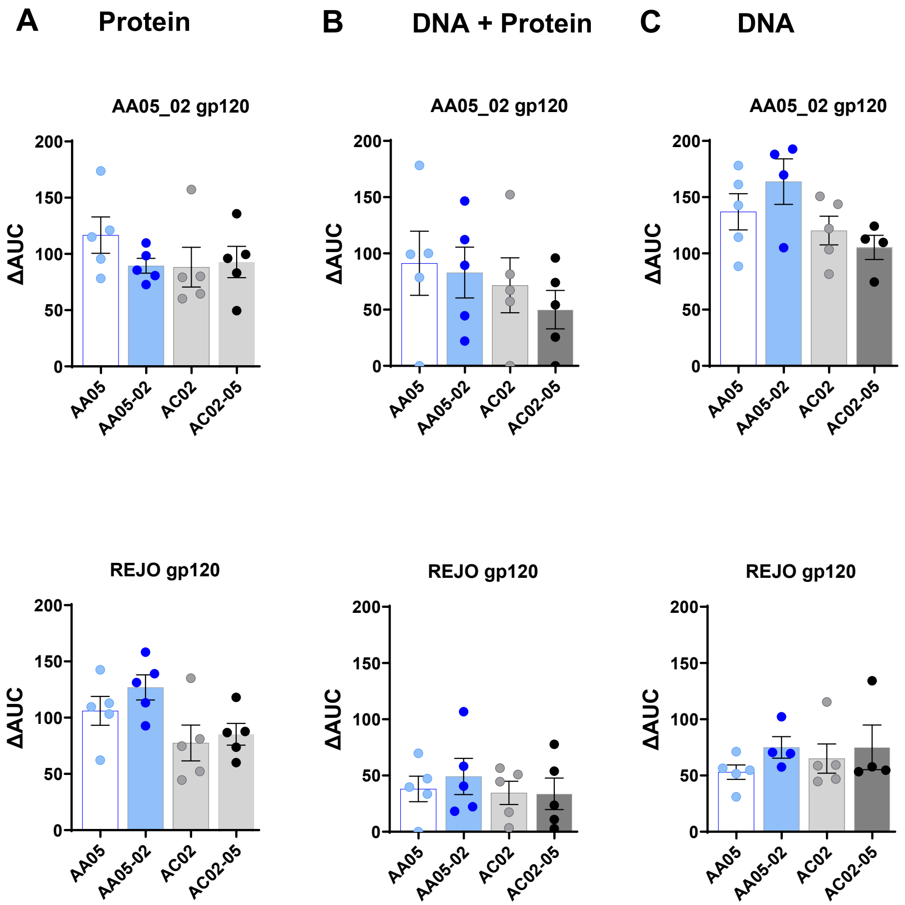

D

ID50 in TZM.bl neutralization assay

Protein Immmunization

Plasma	ID	HXB2	398F1	TRO.11	CH119	RHPA
Control	Pool	<20	<20	<20	<20	NT
AA05	1	<20	<20	<20	<20	NT
	2	<20	<20	<20	<20	NT
	3	<20	<20	<20	<20	NT
	4	<20	<20	<20	<20	NT
	5	<20	<20	<20	<20	NT
AA05-02	1	<20	<20	<20	<20	NT
	2	<20	<20	<20	<20	NT
	3	<20	<20	<20	<20	NT
	4	<20	<20	<20	<20	NT
	5	<20	<20	<20	<20	NT
AC02	1	<20	<20	<20	<20	NT
	2	<20	<20	<20	<20	NT
	3	<20	<20	<20	<20	NT
	4	<20	<20	<20	<20	NT
	5	<20	<20	<20	<20	NT
AC02-05	1	<20	<20	<20	<20	NT
	2	<20	<20	<20	<20	NT
	3	<20	<20	<20	<20	NT
	4	<20	<20	<20	<20	NT
	5	<20	<20	<20	<20	NT

Protein + DNA Immmunization

Plasma	ID	HXB2	398F1	TRO.11	CH119	RHPA
Control	Pool	<20	<20	<20	<20	NT
AA05	1	<20	<20	<20	<20	NT
	2	<20	<20	<20	<20	NT
	3	<20	<20	<20	<20	NT
	4	<20	<20	<20	<20	NT
	5	<20	<20	<20	<20	NT
AA05-02	1	<20	<20	<20	<20	NT
	2	<20	<20	<20	<20	NT
	3	<20	<20	<20	<20	NT
	4	<20	<20	<20	<20	NT
	5	<20	<20	<20	<20	NT
AC02	1	<20	<20	<20	<20	NT
	2	<20	<20	<20	<20	NT
	3	<20	<20	<20	<20	NT
	4	<20	<20	<20	<20	NT
	5	<20	<20	<20	<20	NT
AC02-05	1	<20	<20	<20	<20	NT
	2	<20	<20	<20	<20	NT
	3	<20	<20	<20	<20	NT
	4	<20	<20	<20	<20	NT
	5	<20	<20	<20	<20	NT

Supplementary Figure 3. **Vaccine-Induced antibodies mediate antibody-dependent cellular phagocytosis (ADCP).** Serially diluted samples from **(A)** protein, **(B)** DNA+ Protein and **(C)** DNA immunizations were tested for ADCP using phagocytic THP-1 cells and fluorescent beads coated with AA05_02 and REJO gp120 proteins. Pooled sera from unimmunized animals was tested in parallel as negative control. Titration curves were plotted for ADCP scores and area under titration curve (AUC) were calculated. AUC of negative control sera was subtracted and presented as delta AUC (ΔAUC). (**D**) Serum samples collected from mice immunized with gp120 proteins (left panel) and gp120 proteins + DNA (right panel) immunogens were tested for neutralization of Tier 1 (pseudovirus HXB2) and Tier 2 viruses (pseudovirus 398F1, TRO.11, CH119 and RHPA infectious virus) in a standard assay using the TZM.bl target cells. Pooled serum from unimmunized normal mouse served as negative control (Ctrl). Mouse sera neutralization titers are reported as serum dilution required to inhibit 50% of virus infection (ID50). Reciprocal serum ID50 values that can be measured above the cut-off (1:20) are shown. NT, not tested.

Antigenicity gp120 Proteins

Figure #	Assay	Env Epitopes	Fold change SP swapped/WT (AUC)		
			mAb	AA05-02/AA05	AC02-05/AC02
Fig 1 and 2	ELISA	V1V2	830A	1.94	0.40
			2158	2.01	0.45
			697D	1.19	0.60
			CH58	0.89	2.94
		V3	447	1.43	0.95
			2219	1.65	1.93
		CD4bs	NIH	1.35	0.38
			VRC01	2.06	0.57
		CD4i	17b	0.63	0.30
			A32	1.07	0.51
		Glycan	2G12	1.58	0.21

Antigenicity gp160 DNA

Figure #	Assay	Env Epitopes	Fold change SP swapped/WT (MFI)		
			mAb	AA05-02/AA05	AC02-05/AC02
Fig 3	Flow Cytometry	V1V2	830A	0.75	2.01
			2158	0.63	3.68
			697D	0.75	2.20
			CH59	0.46	2.32
			PG9	0.64	1.88
			PG16	0.77	1.59
			PGT145	0.62	3.87
		V3	447	0.86	1.99
			2219	0.76	2.18
		CD4bs	NIH	1.00	1.70
			VRC01	0.65	2.96
		gp120/gp41 interface	35O22	0.71	2.17
			PGT151	1.09	1.30
		CD4i	A32	1.00	1.78
		Glycan	2G12	1.58	0.21
		MPER	2F5	0.99	1.42

Glycosylation gp120 Proteins (lectin binding)

Figure #	Assay	Env Epitopes	Fold change SP swapped/WT (AUC)		
			mAb	AA05-02/AA05	AC02-05/AC02
Fig 4B	ELISA	T-HM	GNA	1.63	0.57
		HM	NPL	2.49	0.42
		Fucose	AAL	1.22	0.50
		Sialic acid	SNA	0.92	1.06

Glycosylation gp160 DNA (lectin binding)

Figure #	Assay	Env Epitopes	Fold change SP swapped/WT (AUC)		
			mAb	AA05-02/AA05	AC02-05/AC02
Fig 4C	Flow Cytometry	T-HM	GNA	0.65	1.02
		HM	NPL	0.81	0.99
		Fucose	AAL	0.92	0.68
		Gal/Lac	RCA	0.97	0.90
		Sialic acid	SNA	1.00	0.73

Immunogenicity: gp120 Protein

Figure #	Assay	Fold change SP swapped/WT (median)		
		Antigen	AA05-02/AA05	AC02-05/AC02
Fig 5	Luminex	Autologus Ag	8.20	1.62
		Heterologus Ag	1.35	0.96
Fig 8E		V1V2/V3	1.45	1.35
Fig 9A	ADCP	AA05_02 gp120	0.11	0.86
		REJO gp120	1.20	0.60

Immunogenicity: gp120 DNA + gp160 DNA

Figure #	Assay	Fold change SP/WT swapped (median)		
		Antigen	AA05-02/AA05	AC02-05/AC02
Fig 7	Luminex	Autologus Ag	1.24	0.51
		Heterologus Ag	0.93	0.60
Fig 8E		V1V2/V3	0.88	0.77
Fig 9A	ADCP	AA05_02 gp120	0.39	1.17
		REJO gp120	1.01	1.34
Fig 9B	Neutralization	Tier 2 Viruses	0.54	0.96

Immunogenicity: gp120 DNA + gp120 Protein

Figure #	Assay	Fold change SP swapped/WT (median)		
		Antigen	AA05-02/AA05	AC02-05/AC02
Fig 6	Luminex	Autologus Ag	0.23	1.96
		Heterologus Ag	1.51	0.81
Fig 8E		V1V2/V3	0.50	0.38
Fig 9A	ADCP	AA05_02 gp120	3.64	0.05
		REJO gp120	0.45	0.56

Increase by >1 fold, p<0.05

Decrease by >1 fold, p<0.05

Supplementary Figure 4. Summary of the changes in antigenicity, glycosylation and immunogenicity upon SP swapping. Median values and median fold changes of SP swapped/WT were calculated for each parameter. Significant increase and decrease are marked with blue (fold change of >1 with p<0.05) or red (fold change of <1 with p<0.05) respectively. T-HM, terminal high-mannose; HM high-mannose. Fucose, Sialic acid and Galactose (Gal) and Lactose (Lac) are respective sugar moieties on complex glycans. ELISA, Luminex, and flow cytometry assay were used to detect binding Abs and Neutralization and ADCP assays were used to detect functional activities in serum samples.
